# Novel mRNA vaccines induce potent immunogenicity and afford protection against tuberculosis

**DOI:** 10.3389/fimmu.2025.1540359

**Published:** 2025-02-13

**Authors:** Christopher J. De Voss, Marcellus Korompis, Shuailin Li, Alberta Ateere, Helen McShane, Elena Stylianou

**Affiliations:** The Jenner Institute, University of Oxford, Oxford, United Kingdom

**Keywords:** tuberculosis, vaccines, mRNA, viral-vector, BCG

## Abstract

**Introduction:**

*Mycobacterium tuberculosis* (*Mtb*) is the causative agent of tuberculosis (TB), a disease with a severe global burden. The intractability of *Mtb* has prevented the identification of clear correlates of protection against TB and hindered the development of novel TB vaccines that are urgently required. Lipid nanoparticle (LNP)-formulated mRNA is a highly promising vaccine platform that has yet to be thoroughly applied to TB.

**Methods:**

We selected five *Mtb* antigens (PPE15, ESAT6, EspC, EsxI, MetE) and evaluated their potential as LNP-formulated mRNA vaccines, both when each antigen was delivered individually, and when all five antigens were combined in a mix regimen (m-Mix).

**Results:**

Each mRNA construct demonstrated unique cellular and humoral immunogenicity, and both m-Mix, as well as the single antigen EsxI, conferred significant protection in a murine *Mtb* challenge model. Whilst the potent immune responses of each mRNA were maintained when applied as a boost to BCG, there was no additional increase to the efficacy of BCG. Combination of m-Mix with a recombinant, replication-deficient chimpanzee adenovirus (ChAdOx1), in a heterologous prime-boost delivery (C-m-Mix), appeared to result in increased protection upon murine *Mtb* infection, than either regimen alone.

**Discussion:**

This work warrants further investigation of LNP-formulated mRNA vaccines for TB, whilst indicating the potential of m-Mix and C-m-Mix to progress to further stages of vaccine development.

## 1 Introduction

*Mycobacterium tuberculosis (Mtb)* is the etiological agent of tuberculosis (TB), an infectious disease which causes more than 1.3 million deaths annually (1). Chemotherapeutic treatment of TB is challenging, due to the significant course length and associated toxicity, the rise of multi-drug resistant *Mtb* strains, and the geographic distribution of the disease (2–5). The sole licensed vaccine for TB is the live attenuated strain *Mycobacterium bovis* Bacillus Calmette-Guérin (BCG). The efficacy of BCG wanes dramatically during adolescence, and whilst up to 80% of children are protected against disseminated TB by BCG, no significant protection is seen in adults, who comprise 90% of the total cases (1, 6–8). A vaccine with the ability to boost the efficacy of BCG through adulthood would dramatically reduce global TB mortality and be a significant step towards the goal of TB eradication.

The resounding success of lipid nanoparticle (LNP)-formulated mRNA vaccines during the COVID-19 pandemic (9, 10) raised the possibility of extending this promising vaccine platform to other major pathogens, like *Mtb* (11, 12). Due to the difficulty surrounding TB research, particularly the intractability of *Mtb*, the TB vaccine development pipeline has been historically sluggish. Less than 20 TB vaccines are currently in clinical trials (13), compared to more than 180 COVID-19 vaccines that have entered clinical trials since the identification of SARS-CoV-2 (14). The novel mRNA vaccine technology platform presents a safe, adaptable and rapidly scalable option that could provide a fresh and effective option for the TB vaccine pipeline where the few vaccines designed to synergise with BCG are either protein-adjuvant or recombinant viral vectors (13). Such a point is reinforced by the recent commencement of a TB mRNA vaccine phase Ib clinical trial, by BioNTech (NCT05547464). However, very few studies have been published regarding the use of RNA-based vaccine platforms for TB (15–17), and no preclinical or clinical results are available on the efficacy of current-generation LNP-mRNA platforms approved during the COVID-19 pandemic, for TB.

Previous work in our group has identified 4 immunogenic antigens of *Mtb* (PPE15, EspC, EsxI, and MetE), that provide protection against TB in mice when administered as single antigen viral vector (18, 19) or adjuvanted protein vaccines (84) (Almujri, S in preparation). Each of these antigens were produced as an LNP-formulated mRNA vaccine (m-PPE15, m-EspC, m-EsxI, m-MetE). ESAT6 was also produced (m-ESAT6) given its essential role in *Mtb* virulence and numerous previous reports of its immunostimulatory capacity (20–23). As multiple antigens are likely necessary for effective protection against TB (24) in humans, we devised a ‘Mix’ regimen (m-Mix), in which each of the five antigens were delivered together, as a single dose. Notably, PPE15, EsxI, and MetE are expressed in BCG, whilst ESAT6 and EspC are absent or not secreted, respectively (25–27). This varied selection was designed with the intention for our mRNA constructs to boost antigen-specific responses generated by BCG, as well as introduce novel, *Mtb*-specific antigens upon subsequent vaccination.

To our knowledge, this study is one of the first to present preclinical characterisation of the highly successful LNP-mRNA platforms, originally utilised during the COVID-19 pandemic, in specific application against TB. We evaluated 6 novel vaccine regimens for immune parameters that have been associated with protection against TB, as well as through direct efficacy testing by *in vivo* murine TB challenge studies. Our findings indicate strong humoral and cellular immunogenicity to the antigens, as well as protective efficacy for two mRNA regimens: m-EsxI and m-Mix. Furthermore, we explored the potential of heterologous prime-boost strategies using intranasal ChAdOx1 viral vectors, to enhance protective mucosal immune responses, whilst harnessing the immunogenic properties of the mRNA platform. We identified effective synergy of m-Mix with the ChAdOx1 platform, supporting progression of this vaccine candidate to the next stage of development, as well as further investigation of mRNA technology against TB.

## 2 Methods

### 2.1 Mice and vaccinations

Female, 6–8-week-old CB6F1 mice, were purchased from Charles River Germany. All procedures were performed in accordance with the UK Animals (Scientific Procedures) Act 1986, under project license number P9804B4F1, granted by the UK Home Office. Animal studies were approved by the Animal Welfare and Ethical Review Board (AWERB), University of Oxford. Administration of all substances was performed under short-term anaesthesia, using vaporised IsoFlo^®^. mRNA vaccines were delivered intramuscularly to the right leg (50 μL), at either 1 μg or 5 μg per dose. For the m-Mix regimen, 1 μg of each of the 5 constructs were combined immediately prior to injection. 1x10^8^ infectious units (ifu) of recombinant ChAdOx1 viral vectors were administered drop-by-drop to the nostrils (30 μL), to access the intranasal route. BCG Pasteur (ATCC 35734) was cultured at 37°C in Middlebrook 7H9 broth, supplemented with 0.05% v/v tween-80 and 10% v/v ADC enrichment (Sigma-Aldrich) or on Middlebrook 7H11 agar supplemented with 0.5% v/v glycerol and 10% v/v OADC enrichment (BD Bioscience). BCG was administered via the intradermal route to the ear, at 3x10^5^ colony forming units (CFU) per dose (50 μL). At experimental endpoints, animals were euthanised by cervical dislocation.

### 2.2 LNP-formulated mRNA production

mRNA encoding each of the 5 antigens was codon-optimised for mammalian expression and synthesised *in vitro* using an optimised T7 RNA polymerase-mediated transcription reaction, with complete replacement of uridine by N1-methyl-pseudouridine (28). The reaction included a DNA template containing the antigen open reading frame flanked by 5′ untranslated region (UTR) and 3′ UTR sequences and was terminated by an encoded polyA tail. The 5’ and 3’ UTR sequences, cap1, and polyA tail were engineered to improve stability and translational efficiency of the mRNA. The mRNA was purified by oligo-dT affinity purification, buffer exchanged, sterile filtered, and kept frozen at –20°C until further use. The mRNA was encapsulated in LNPs through a modified ethanol-drop nanoprecipitation process, as described previously (29). Lipids in the LNP include SM-102 (a custom manufactured, ionizable lipid), PEG2000-DMG, cholesterol, and DSPC. These ionisable, structural, helper, and polyethylene glycol lipids were combined with prepared mRNA in acetate buffer (pH 5.0), at a 5:2 ratio of lipids:mRNA. The mixture was neutralised with tris-Cl (pH 7.5), combined with sucrose as a cryoprotectant, sterile-filtered, and stored at −70°C until further use. The product underwent analytical characterisation, which included the determination of particle size and polydispersity, pH, osmolarity, endotoxin, mRNA encapsulation, purity, and concentration, before the material was deemed acceptable for *in vivo* studies.

### 2.3 *In vitro* mRNA expression

HEK293 cells were transiently transfected with 100 ng/μL of mRNA in Opti-MEM (Gibco), using Lipofectamine 2000 (Invitrogen). 24 hours after transfection, protein samples were obtained using the RIPA Lysis Buffer system (Santa Cruz Biotechnology) and visualised by SDS-PAGE on NuPAGETM 4 to 12%, Bis-Tris, 1.5 mm Protein Gels (Invitrogen). For western blotting, proteins were transferred onto polyvinylidene difluoride (PVDF) membrane using a Trans-Blot^®^ TurboTM (Bio-Rad) and blotting performed using the iBind system (Life Technologies). Blots were first probed with polyclonal sera (1:1000 dilution) obtained from mice vaccinated with relevant purified protein mixed in Quil-A (InvivoGen) or AS01 (Shingrix). Purified PPE15, ESAT6, EsxI, and EspC were produced by BiologicsCorp, USA, and purified MetE was obtained from GenScript, USA. An anti-mouse IgG-Alkaline Phosphatase antibody produced in goat (Sigma-Aldrich) (1:1000 dilution) allowed visual detection, in conjunction with BCIP/NBT tablets (Sigma-Aldrich), according to manufacturer’s instructions.

### 2.4 Viral vector creation

Generation of ChAdOx1.EsxI has been described previously (19) and was followed for ChAdOx1.15-3E (C-Mix). Briefly, the antigen sequence of 15-3E from *Mtb* was codon-optimised for mammalian expression, with an N- terminal signal peptide from human tissue plasminogen activator (Thermo-Fisher). No linker sequences were inserted between the coding regions of the antigens in 15-3E. Constructs were cloned into a Gateway entry plasmid (Thermo-Fisher), under control of the human cytomegalovirus immediate-early promoter, prior to recombination into a ChAdOx1 destination vector. The vector was linearised and transfected into HEK293 cells, prior to viral purification by CsCl gradient ultracentrifugation, performed at the Viral Vector Core Facility, University of Oxford.

### 2.5 Flow cytometry

Splenocytes were obtained by mashing. Lungs were perfused with PBS, prior to being cut into small pieces and incubated with DNase and collagenase (Sigma-Aldrich). Resultant suspensions from both organs were treated with ACK lysis buffer (150 mM NH_4_Cl, 10 mM KHCO_3_, 0.1 mM Na_2_EDTA, pH 7.2-7.4) and resuspended in DMEM (Sigma-Aldrich). Cells were stimulated for 2 hours (37°C), with purified protein derivative (PPD) (10 μg/mL) (AJ Vaccines), MetE whole protein (10 μg/mL) (GenScript), or 15-mer peptide pools (2 μg/mL), overlapping by 11 amino acids, for the entire sequence of ESAT6, EspC, or EsxI, or the N-terminal 19kDa of PPE15 (Peptide Synthetics, UK). Following stimulation, GolgiPlug (BD Bioscience) was added (1 μL/mL) and cells were incubated for a further 4 hours (37°C), before being stored at 4°C overnight. Cells were then stained with LIVE/DEAD fixable red dead cell stain (Invitrogen) (10 min, 4°C), before incubation with anti-CD16/32 and a panel of surface markers comprising anti-CD45R, -TCRαβ, -TCRγδ, -CD4, and -CD8 (eBioscience) (30 min, 4°C). Thereafter, cells were fixed and washed using CytoFix and Perm/Wash (BD Bioscience), before incubation with anti-IFNγ, -TNFα, -IL-2, -IL-17A (eBioscience) (30 min, 4°C). Stained cells were immediately run on an LSR II Flow Cytometer (BD Bioscience).

### 2.6 Enzyme-linked immunosorbent assay

Serum was obtained from blood collected by cardiac puncture. Nunc Maxisorp 96-well plates (ThermoFisher) were coated overnight with 2 μg/mL of PPE15, ESAT6, EspC, or EsxI protein (BiologicsCorp, USA), or MetE protein (GenScript). Plates were washed with PBST (PBS + 0.05% v/v Tween20) before blocking with PBS + 2.5% w/v Bovine Serum Albumin (BSA) (90 min). Sera samples were diluted 1/50 in PBST + 0.01% w/v BSA. Samples were then serially diluted 5-fold a further 6 times, for 7 total dilutions, and incubated for a further 90 min (room temperature). Plates were washed, before addition of alkaline phosphatase-conjugated goat anti-mouse IgG (1/5000) (BioRad). After incubation for 1 hour, plates were washed and developed with 4-nitrophenyl phosphate (1 mg/mL) (Sigma-Aldrich) diluted in diethanolamine buffer (ThermoFisher). Optical Density 405 nm was measured using a BioTeK microplate spectrophotometer (Gen5 software).

### 2.7 Enzyme-linked ImmunoSpot

Millipore Multi-screen Immobilon-P membrane plates (Merck) were coated overnight (4°C) with rat anti-mouse IFNγ mAB AN18 (Mabtech) (5 μg/mL), diluted in carbonate-bicarbonate buffer (0.05 M, pH 9.6). Plates were then blocked with DMEM (>1 hour, 37°C). Isolated splenocytes were plated at 5x10^5^ or 2.5x10^5^ cells per well, with relevant peptide pool (2 μg/mL) stimulation, if required. Plates were incubated for 18-20 hours (37°C), after which they were washed with PBS. Biotinylated anti-mouse IFNγ mAB R4-6A2 (Mabtech) was then added (1 μg/mL) for 2 hours (room temperature). Plates were washed before incubation with Streptavidin Alkaline Phosphatase (Mabtech) (1 μg/mL) for 1 hour (room temperature). Plates were washed again before development with AP substrate conjugate kit (BioRad). Spots were counted using an ELISpot reader system ELR02 (AID Diagnostika).

### 2.8 *Mtb* infection studies

Aerosol *Mtb* infections were performed using the Biaera AeroMP-controlled nebulizer (Biera Technologies, Hagerstown, MD, USA), within a biosafety level 3 Total Containment Oxford Ltd isolator. Mice were placed in nose-only restrainers and infected with aerosolised *Mtb* Erdman K01 (TMC107; BEI Resources, Manassas, VA, USA). Lung infection burdens were verified in 2 animals, 1-hour post-challenge (average 50 CFU). Four weeks post-challenge, lung and spleens were harvested and homogenised using a Precellys 24 homogenizer (Stretton Scientific). Serial dilutions were prepared in PBS, prior to plating on modified Middlebrook 7H11 (Animal and Plant Health Agency, UK). Plates were incubated at 37°C and counts obtained after 3-4 weeks.

### 2.9 Data analysis

FloJo v10.10 was used to analyse flow cytometry data, following a defined gating strategy (**Supplementary Figure 1**). Pestle v2 and SPICE v6.1 were used for multifunctional cytokine analysis. GraphPad Prism v10.2 was used for graph preparation and statistical analysis. Statistical analysis was conducted using Kruskal-Wallis one way ANOVA with Dunn’s test for multiple comparisons. Significance was considered when p<0.05.

## 3 Results

### 3.1 Novel TB mRNA vaccines induce strong cellular and humoral systemic immune responses

**Figure 1:**
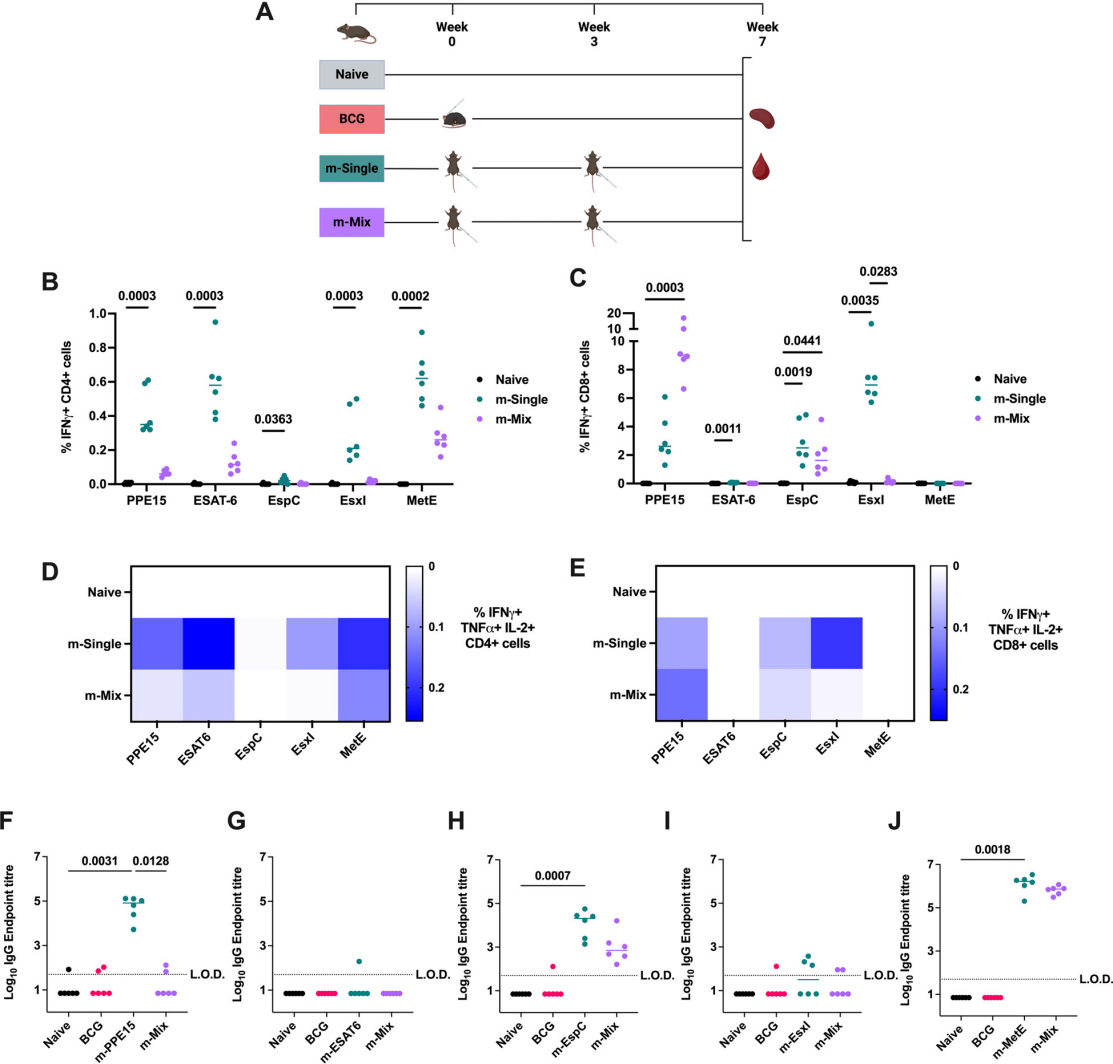
Antigen-specific immunogenicity of novel TB mRNA vaccines. **(A)** Immunisation schedule, created with BioRender.com. Groups of CB6F1 mice were vaccinated twice with one of five single antigen mRNA vaccines (m-Single) administered at 5 μg per dose, or an equal mix of all 5 antigens (m-Mix) for the same total dose (1 μg each antigen). Immune responses in the spleen and blood were quantified four weeks post-boost. **(B, C)** Flow cytometric analysis of IFNγ expression by **(B)** CD4+ T cells or **(C)** CD8+ T cells in the spleen, in response to stimulation by relevant antigens listed on x-axis. For clarity, only statistically significant comparisons are shown. **(D, E)** Heatmaps demonstrate the proportion of triple cytokine-secreting IFNγ+ TNFα+ IL-2+ **(D)** CD4+ or **(E)** CD8+ T cells, in response to stimulation by antigens listed on horizontal axis. **(F-J)** Sera was analysed by ELISA for endpoint IgG titres to **(F)** PPE15, **(G)** ESAT6, **(H)** EspC, **(I)** EsxI, or **(J)** MetE. L.O.D. indicates “limit of detection” for minimum calculable endpoint titre; values under L.O.D. were arbitrarily assigned half the L.O.D. value. Each symbol represents response from 1 animal, n=6 per group. **(B, C, F-J)** Horizontal bars, or **(D, E)** colour intensity, indicate median. Statistical significance was determined via Kruskal-Wallis ANOVA with Dunn’s test for multiple comparisons, selected comparisons displayed only.

To evaluate the immune response induced by each construct, CB6F1 mice were administered a single antigen mRNA vaccine (m-Single) at 5 μg per dose, or an equal ‘mix’ of all 5 antigens (m-Mix) with each antigen contributing 1 μg, for a total dose of 5 μg (**Figure 1A**). Antigen-specific immune responses were assessed by IFNγ, TNFα, IL-2, and IL-17 production, given their association with TB protection (30, 31). Significant CD4+ T cell responses were observed in the spleen toward all single antigen regimens, as demonstrated by high production of IFNγ (**Figure 1B**), TNFα, and IL-2, but not IL-17 (**Supplementary Figures 2A, C, E**) (**Supplementary Table 1**). The IFNγ response to EspC was relatively low, albeit statistically significant in m-EspC compared to the naïve group (p=0.0363). For PPE15, ESAT6, and MetE, the antigen-specific responses in m-Mix were higher than those in the naïve group, although decreased relative to the m-Single regimens. However, for none of the antigens was this reduction by a statistically significant degree (largest decrease for EsxI, p=0.1179). Measurement of the CD8+ T cell response demonstrated very strong IFNγ responses to PPE15, EsxI, and EspC (**Figure 1C**), in addition to TNFα, and IL-2 (**Supplementary Figures 2B, D, F**). In m-Mix, these responses were equivalent for EspC but were significantly reduced for EsxI (p=0.003). The PPE15-specific CD8+ T cell response trended toward an increase in m-Mix (p=0.1508), relative to the m-PPE15 group. Very low, although significant, CD8+ T cell responses were observed to ESAT6 (p=0.0011) (for clarity, see **Supplementary Figures 3A, B** and **Supplementary Table 1**). Triple cytokine-secreting antigen-specific CD4+ T cells, i.e. IFNγ+ TNFα+ IL-2+, were strongly induced towards PPE15, ESAT-6, and MetE in the m-Single regimens, and remained present in the m-Mix group (**Figure 1D**). Moreover, the proportion of other polyfunctional subtypes observed in m-Single, i.e. expressing various combinations of IFNγ, TNFα, and IL-2, also remained consistent in m-Mix (**Supplementary Figures 2G, H**). Triple positive CD8+ T cells were detectable to PPE15, EspC, and EsxI (**Figure 1E**); however, the majority of multifunctional CD8+ T cells were either: IFNγ+ TNFα- IL-2-, or IFNγ+ TNFα+ IL-2- (**Supplementary Figures 2H, I**).

Given the potentially significant role for antibodies in protective immunity against *Mtb* (32), sera was collected at 4 weeks post-boost. Robust IgG endpoint titres (**Figures 1F–J**) were detected toward PPE15, EspC, and MetE in the respective m-Single groups. In the m-Mix group, the responses to EspC and MetE were statistically equivalent to those in the m-Single groups. However, no significant PPE15 IgG was detected in the m-Mix group, and both ESAT6 and EsxI induced low humoral responses (**Figures 1G, I**). These results align with *in vitro* transfections of HEK293 cells using these mRNA, which demonstrated expression of PPE15 and MetE, but no detectable ESAT6, EspC, or EsxI (**Supplementary Figure 4**) (summarised in **Supplementary Table 1**).

### 3.2 Changes in PPE15- and EsxI-specific responses in m-Mix are likely not due to dose reduction

The modest reduction in antigen-specific responses between each m-Single and the m-Mix group was expected, due to the dosing difference (i.e., 5 μg in m-Single vs 1 μg per antigen in m-Mix). However, the trends for PPE15 and EsxI were notably different. To determine whether the pronounced reduction in EsxI-specific cellular responses and the trend toward increased PPE15-specific CD8+ T cell response in m-Mix group were due to the lower mRNA dose, a separate immunogenicity study was conducted. In this study, animals received either the m-Single regimens (i.e., m-PPE15 5μg or m-EsxI 5μg), or m-Mix regimen (1μg of each of the 5 antigens). In addition, two groups received the same dose of PPE15 or EsxI as in m-Mix regimen, but each antigen was administered individually (i.e. m-PPE15 1μg or m-EsxI 1μg).

**Figure 2:**
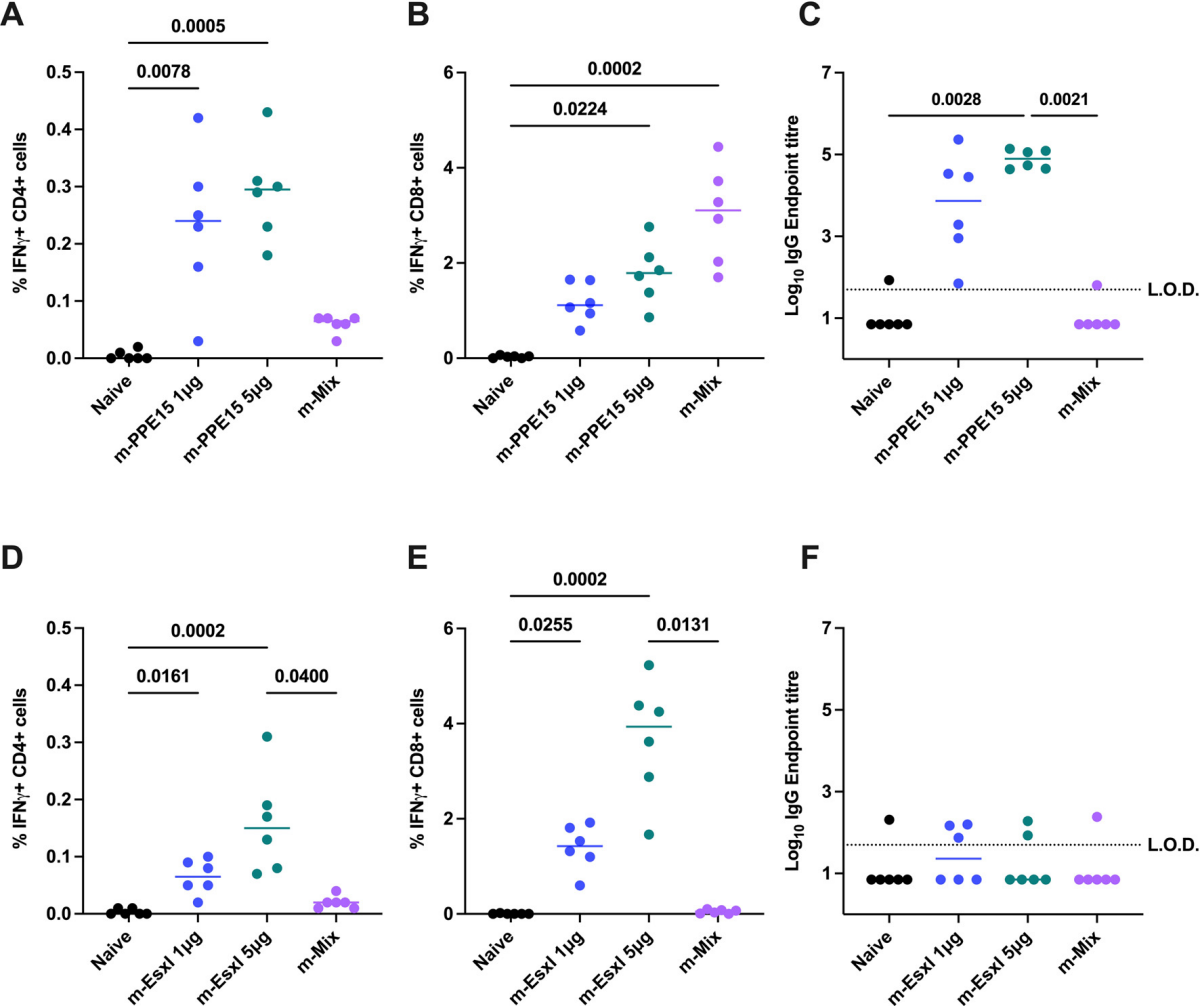
Impact of mRNA dose on PPE15- and EsxI-specific responses in m-Single compared to m-Mix regimen. Groups of CB6F1 mice were vaccinated twice with 1 μg or 5 μg of m-PPE15 or m-EsxI, or an equal mix of all 5 antigens (m-Mix) (5 μg total). Immune responses were quantified in the spleen and blood four weeks post-boost. **(A, B)** Flow cytometric analysis of IFNγ expression by **(A)** CD4+ or **(B)** CD8+ T cells, in response to PPE15 stimulation. **(D, E)** As for **(A, B)**, except EsxI. **(C, F)** Sera was analysed by ELISA for endpoint IgG titres to **(C)** PPE15 or **(F)** EsxI. L.O.D. indicates “limit of detection” for minimum calculable endpoint titre; values under L.O.D. were arbitrarily assigned half the L.O.D. value. Each symbol represents response from 1 animal, n=6 per group. Horizontal bars indicate median. Statistical significance determined via Kruskal-Wallis ANOVA with Dunn’s test for multiple comparisons, selected comparisons displayed only.

In agreement with previous data, the relationships between m-Mix and m-EsxI 5μg, or m-PPE15 5μg, were replicated. The m-PPE15 1μg condition elicited a stronger CD4+ T cell response (p=0.7764) (**Figure 2A**) and higher levels of IgG (p=0.0702) (**Figure 2C**), compared to the PPE15-specific response in the m-Mix group, albeit with group variability. There was a trend for lower CD8+ T cell responses in the m-PPE15 1μg group (**Figure 2B**), compared to the m-Mix group (p=0.0958).

Distinct differences were also seen between m-EsxI 1μg and the m-Mix regimen. Delivery of m-EsxI 1μg induced CD4+ (**Figure 2D**) and CD8+ (**Figure 2E**) T cell responses that were greater than those observed in the m-Mix condition, although not statistically significant. Minimal EsxI-specific IgG was observed (**Figure 2F**). These results provide evidence that the variations in PPE15- and EsxI-specific responses in the m-Mix group, relative to the 5μg single antigen delivery, were likely not due to dose alone.

### 3.3 m-EsxI and m-Mix formulations protect against TB in a mouse model

**Figure 3:**
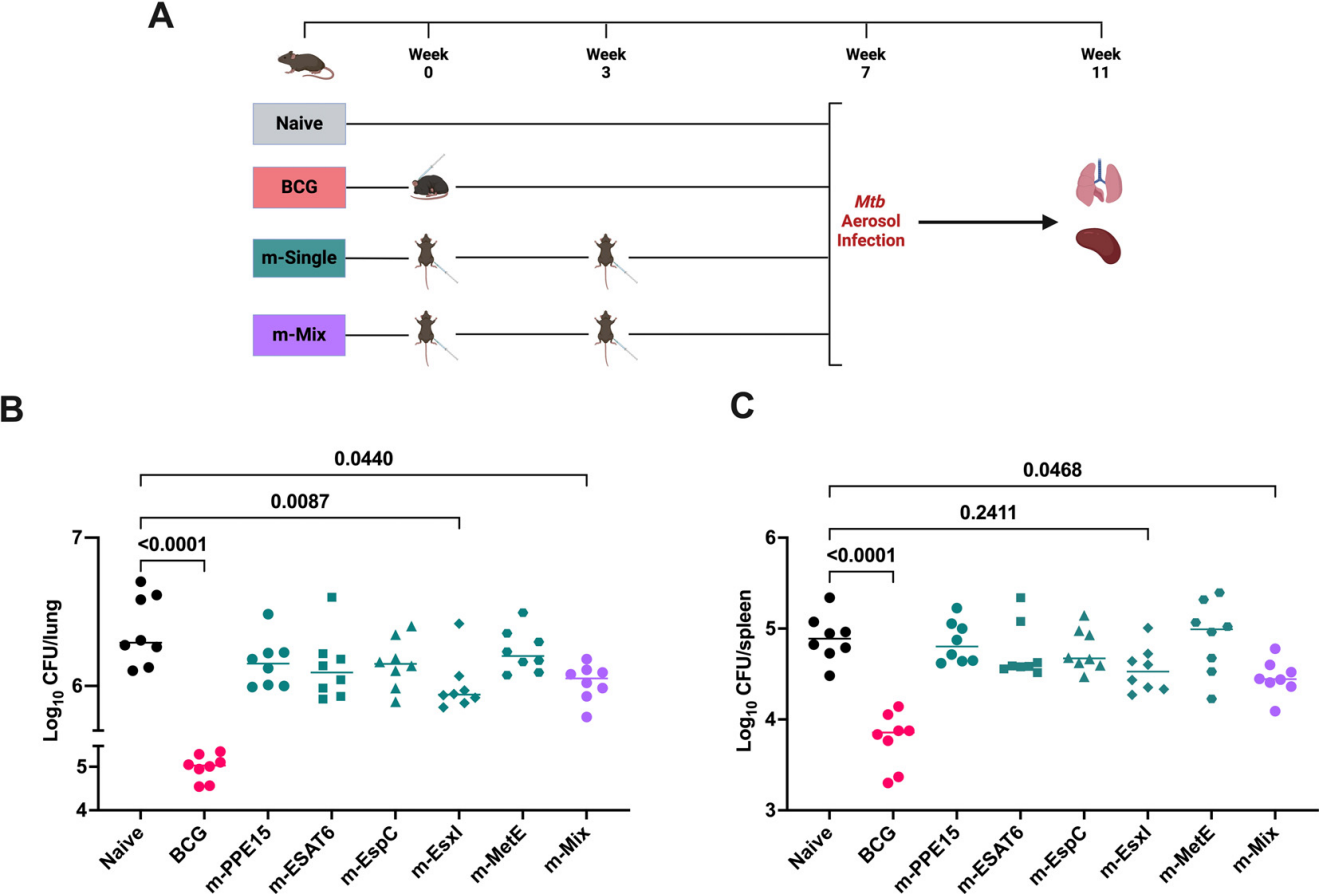
Protective efficacy of novel TB mRNA vaccines against aerosol *Mtb* infection. **(A)** Immunisation schedule and experimental schematic, created with BioRender.com. Mice vaccinated with m-Single (5 μg), or m-Mix (5 μg total), were infected 4 weeks post-boost, whilst BCG-vaccinated mice were infected 7 weeks post-vaccination. **(B, C)** *Mtb* colony forming units (CFU) in the **(B)** lungs and **(C)** spleen of CB6F1 mice, 4 weeks after infection with low-dose aerosol *Mtb.* Each symbol represents the bacterial load in 1 animal, n=8 per group. Horizontal bars indicate median. Statistical significance determined via Kruskal-Wallis ANOVA with Dunn’s test for multiple comparisons, selected comparisons displayed only.

The strong cellular responses and the induction of specific IgG following mRNA vaccination, led us to evaluate vaccine efficacy *in vivo*. Mice were vaccinated with either an m-Single (5 μg) regimen or m-Mix (5 μg total) for comparison against unvaccinated mice. BCG-vaccinated animals were included in this experiment as a positive control (**Figure 3A**). Following vaccination, mice were infected with low-dose aerosol *Mtb*. Four weeks post-challenge, the lung and spleen were collected for bacterial enumeration. BCG vaccination induced a significant level of protection compared to the naïve control group, in both organs (p<0.0001). There was also a significant reduction in lung bacterial burden in the m-EsxI (p=0.0087) and the m-Mix (p=0.044) groups, and most single antigens also appear to trend towards a CFU reduction compared to the naïve control group (**Figure 3B**). Similar trends were observed in the spleen, but only the m-Mix (p=0.0468) reached statistical significance when compared to unvaccinated naïve animals (**Figure 3C**).

### 3.4 TB mRNA vaccines maintain strong systemic immunogenicity when administered as a boost to BCG

Given that real-world clinical dosing would deliver a novel mRNA vaccine as a boost to BCG administration in infanthood, we sought to evaluate the immunogenicity of our candidates in a historically BCG-primed background. Mice were vaccinated intradermally with BCG, and after 10 weeks rest, received two doses of an m-Single vaccine (5 μg), or m-Mix (5 μg total). Four weeks after the final booster vaccination, systemic immunogenicity was evaluated in comparison to naïve and BCG-only groups.

**Figure 4:**
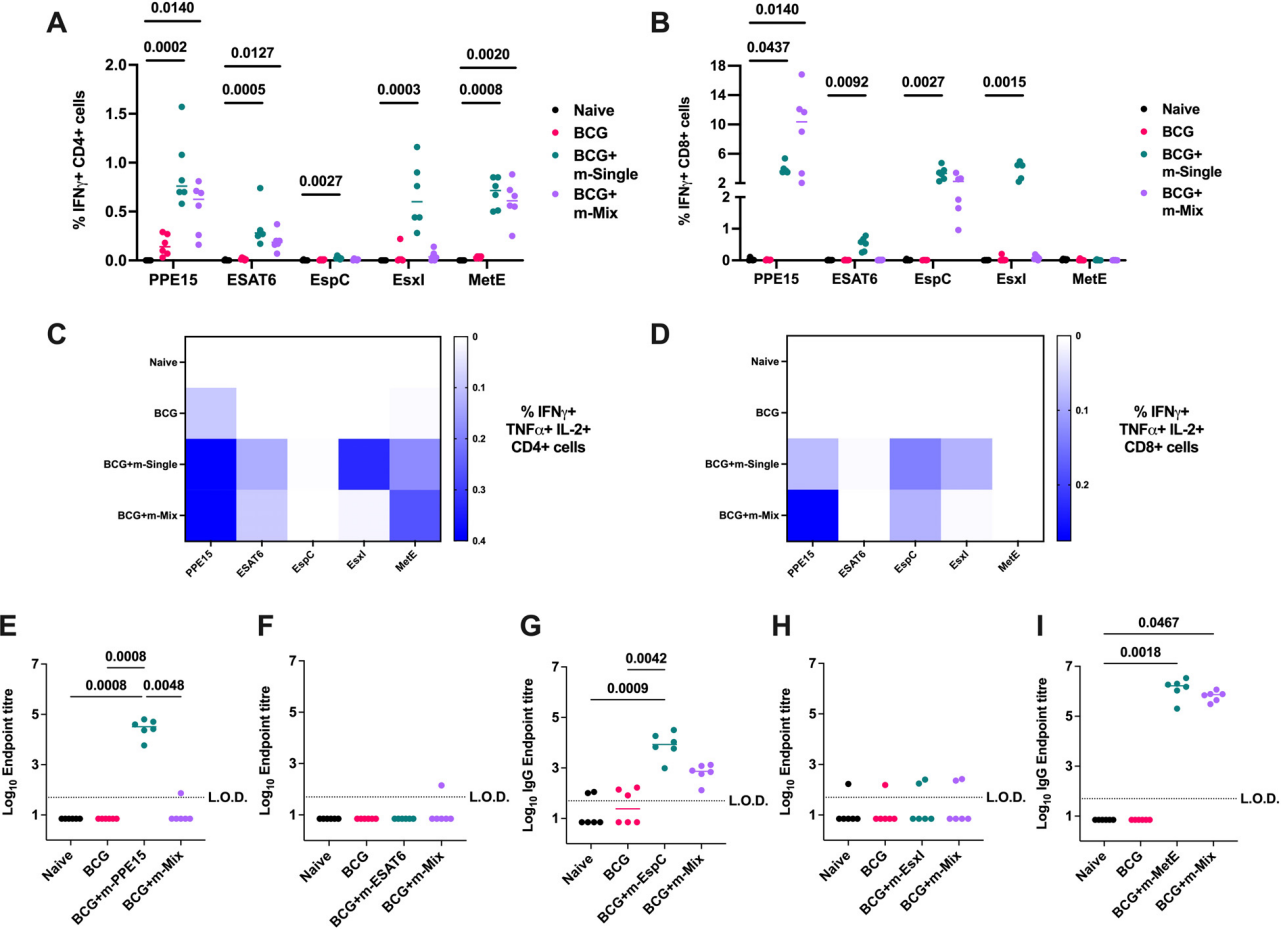
Evaluation of TB mRNA vaccine immunogenicity when applied as a boost to BCG. Groups of CB6F1 mice were vaccinated with BCG, and after 10 weeks rest, relevant groups were vaccinated twice with single antigen mRNA vaccines (m-Single) or an equal mix of all 5 antigens (m-Mix). Four weeks post-boost, immune responses in the spleen and blood of all animals were quantified. **(A, B)** Flow cytometric analysis of IFNγ expression by **(A)** CD4+ or **(B)** CD8+ T cells in the spleen, in response to stimulation by relevant antigens listed on x-axis. For clarity, only significant statistical comparisons between the naïve group and m-Single groups, or m-Mix, are shown. **(C, D)** Heatmaps demonstrate the proportion of triple polypositive IFNγ+ TNFα+ IL-2+ **(C)** CD4+ or **(D)** CD8+ T cells, in response to stimulation by antigens listed on horizontal axis. **(E-I)** Sera was analysed by ELISA for endpoint IgG titres to **(E)** PPE15, **(F)** ESAT6, **(G)** EspC, **(H)** EsxI, or **(I)** MetE. L.O.D. indicates “limit of detection” for minimum calculable endpoint titre; values under L.O.D. were arbitrarily assigned half the L.O.D. value. Each symbol represents response from 1 animal, n=6 per group. **(A, B, E-I)** Horizontal bars, or **(C, D)** colour intensity, indicate median. Statistical significance determined via Kruskal-Wallis ANOVA with Dunn’s test for multiple comparisons, selected comparisons displayed only.

All vaccine groups had robust and equivalent CD4+ responses to PPD (**Supplementary Figure 5A**). Quantification of vaccine antigen-specific responses indicated strong CD4+ T cell production of IFNγ (**Figure 4A**), TNFα, and IL-2 (**Supplementary Figures 5B, C**), for all m-Single regimens except m-EspC, which induced weaker, yet still significant responses compared to the naïve group (p=0.0027). These responses were maintained in the m-Mix group for PPE15, ESAT-6, and MetE, but were more greatly reduced for EsxI (p=0.1051). Robust antigen-specific CD8+ T cell responses were detected in the m-PPE15, m-EspC, m-EsxI and m-ESAT6 groups, compared to the naïve cohort. In the m-Mix group, there was a trend towards an increased PPE15-specific CD8+ T cell response for m-Mix, compared to m-PPE15 (**Figure 4B**) (**Supplementary Figures 5D, E**), whilst the EsxI-specific response trended towards a reduction in m-Mix, relative to the m-EsxI group (p=0.2301). The EspC-specific response was comparable between m-EspC and m-Mix.

A high proportion of multifunctional IFNγ+ TNFα+ IL-2+ CD4+ and CD8+ T cells was observed for PPE15 in both the single antigen and m-Mix groups. Additionally, robust triple-positive CD4+ T cell responses were detected for EsxI and MetE in the single antigen regimens (**Figures 4C, D**). Analysis of humoral immunity (**Figures 4E–I**) revealed high IgG titres to PPE15, EspC, and MetE in the single antigen regimens. Similar responses were observed in the m-Mix group; however, no PPE15-specific IgG was detected.

### 3.5 Novel mRNA vaccine candidates do not improve the efficacy of BCG in a mouse model

**Figure 5:**
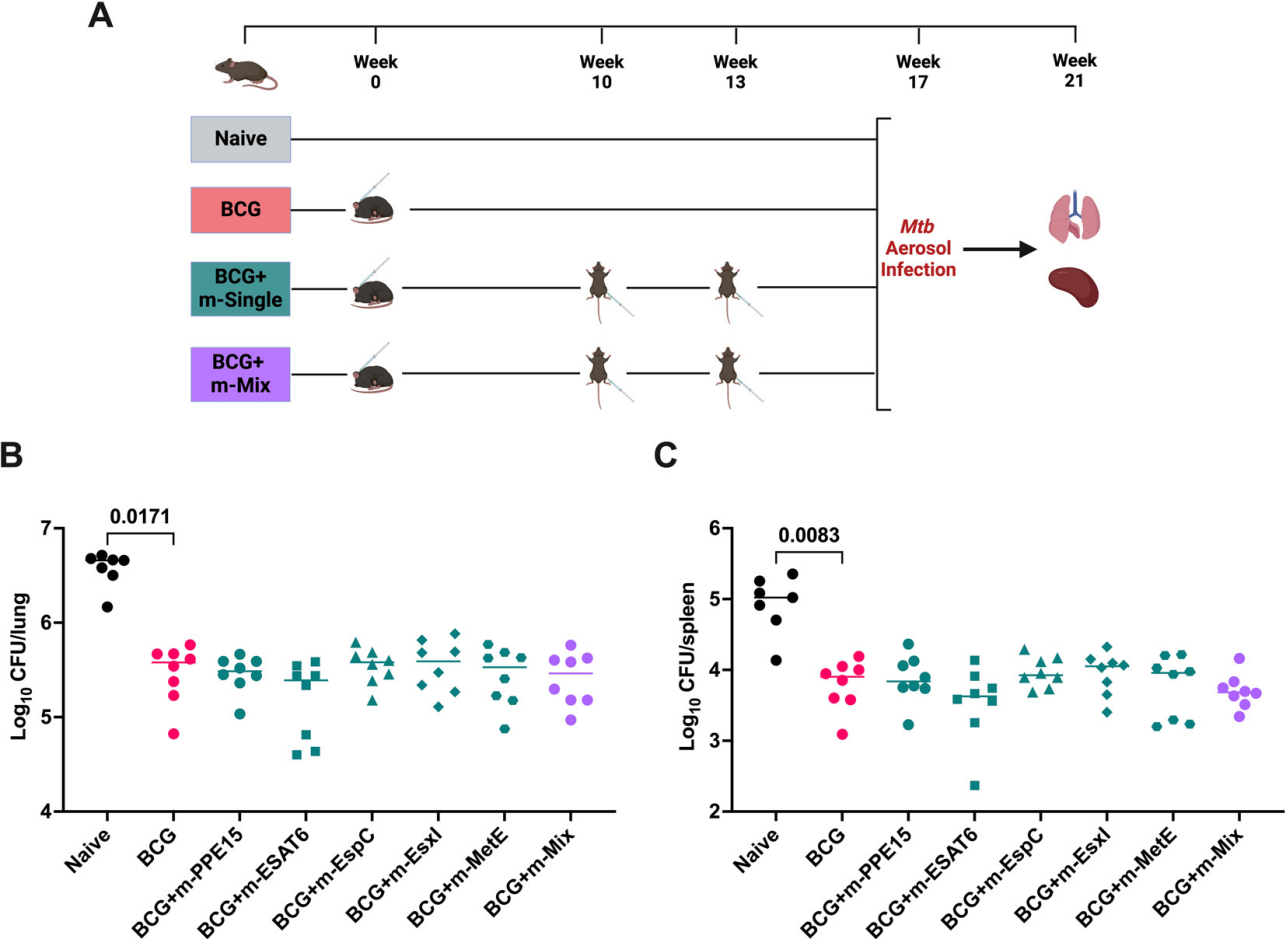
Protective efficacy of novel TB mRNA vaccines, when delivered as a boost to BCG. **(A)** Immunisation schedule and experimental schematic, created with BioRender.com. BCG-vaccinated mice were allowed 10 weeks rest, before boosting with two doses of m-Single (5 μg), or m-Mix (5 μg total). Infections were performed 4 weeks post-boost, or 17 weeks post-BCG vaccination. **(B, C)** *Mtb* colony forming units (CFU) in the **(B)** lungs and **(C)** spleen of CB6F1 mice, 4 weeks after challenge with low-dose aerosol *Mtb*. For clarity, only the statistical comparison between naïve and BCG groups are shown, although all mRNA vaccine groups were significantly reduced relative to naïve. Each symbol represents response from 1 animal, n=8 per group. Horizontal bars indicate median. Statistical significance determined via Kruskal-Wallis ANOVA with Dunn’s test for multiple comparisons.

Given the maintenance of strong antigen-specific responses in BCG-vaccinated animals, the efficacy of the mRNA platform was evaluated in historically BCG-vaccinated mice (**Figure 5A**). All BCG-vaccinated groups were afforded significant protection in the lung relative to unvaccinated mice (p-values not shown on graph), and the vast majority in the spleen, aside from BCG+m-EspC (p=0.1233) and BCG+m-EsxI (p=0.1029). However, there was no significant further increase in protection for any of the mRNA-boosted groups, relative to BCG-only (**Figures 5B, C**). Modest trends towards reduced infection burden in the spleen may be seen in the BCG+m-ESAT6 and BCG+m-Mix groups.

### 3.6 Heterologous prime-boost vaccination with a viral vector and mRNA stimulates strong systemic cellular responses and antibody production

**Figure 6:**
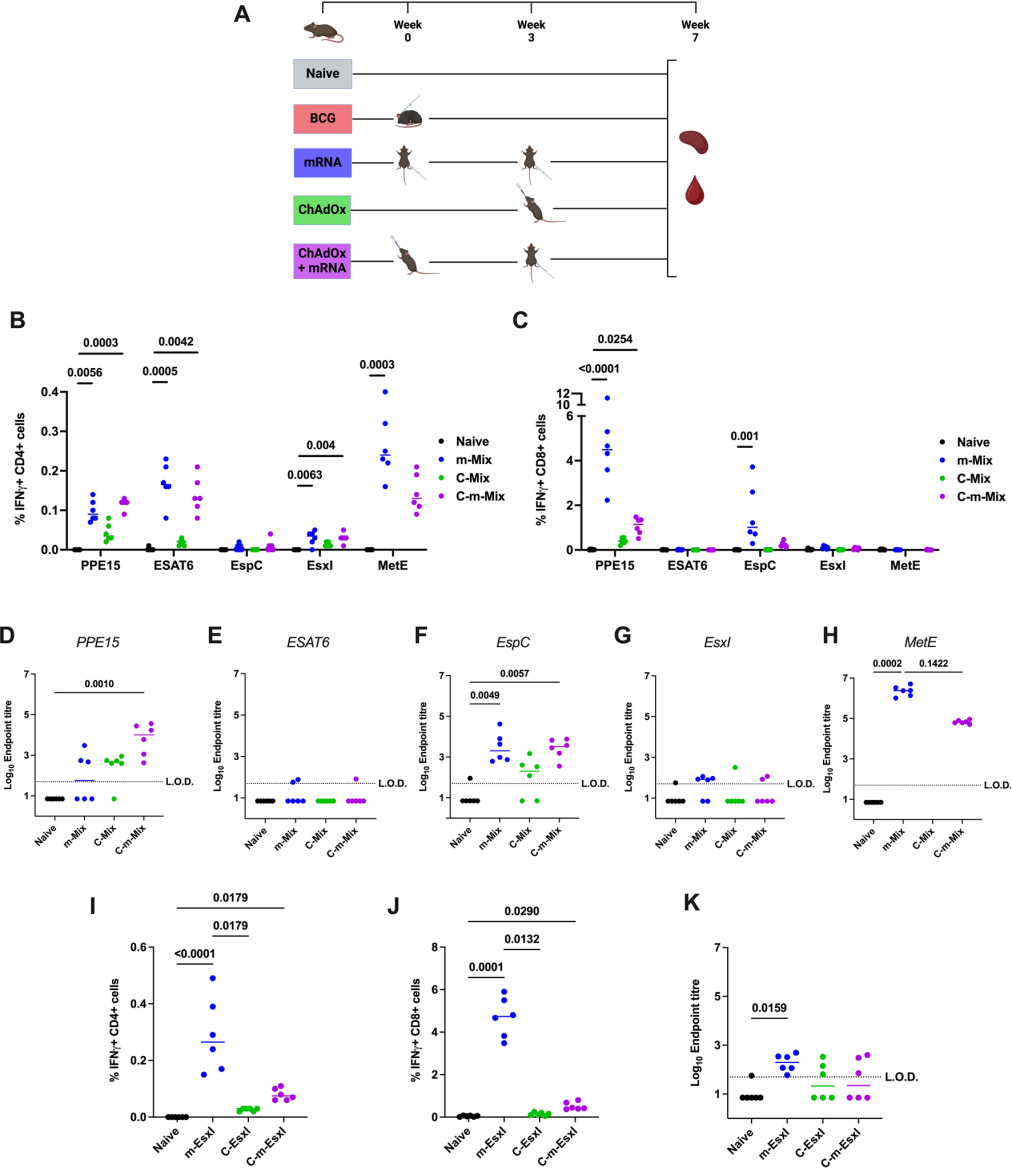
Systemic immune responses following heterologous ChAdOx-mRNA prime-boost. **(A)** Immunisation schedule, created with BioRender.com. Groups of CB6F1 mice were vaccinated twice with mRNA (‘m’), once with ChAdOx1 (‘C’), or with one dose of ChAdOx followed by a booster dose of mRNA (‘C-m’), in either a Mix or EsxI experimental arm. Four weeks post-boost, immune responses in the spleen and blood of all animals were quantified. **(B, C)** Flow cytometric analysis of IFNγ expression by **(B)** CD4+ or **(C)** CD8+ T cells in the spleens of animals in Mix groups, in response to stimulation by relevant antigens listed on x-axis. For clarity, only significant comparisons between the naïve and m-Mix or C-m-Mix groups are shown. **(D-H)** Sera from groups in the Mix category was analysed by ELISA for endpoint IgG titres to **(D)** PPE15, **(E)** ESAT6, **(F)** EspC, **(G)** EsxI, or **(H)** MetE. L.O.D. indicates “limit of detection” for minimum calculable endpoint titre; values under L.O.D. were arbitrarily assigned half the L.O.D. value. **(I-K)** Flow cytometric analysis of IFNγ expression by **(I)** CD4+ T cells or **(J)** CD8+ T cells in the spleens of animals in EsxI groups, in response to stimulation by EsxI peptide pool. **(K)** Sera from groups in the EsxI category was analysed by ELISA for endpoint IgG titres to EsxI. Each symbol represents the response from 1 animal, n=6 per group. Horizontal bars indicate median. Statistical significance determined via Kruskal-Wallis ANOVA with Dunn’s test for multiple comparisons, selected comparisons displayed only.

With no clear boosting effect of mRNA on the efficacy of BCG, we sought to design a heterologous vaccine combination that may enhance the ability of mRNA to confer protection in the lung, as the primary infection site of *Mtb*. To this end, the ChAdOx1 viral vector was utilised, given its previous success in generating significant TB antigen-specific lung responses (18, 19). m-EsxI and m-Mix were selected for the two arms of this trial, given their protective efficacy observed in this study (**Figure 3**). We designed a four-antigen fusion of PPE15-ESAT6-EspC-EsxI (15-3E), to mimic delivery of the multi-antigen m-Mix regimen, thus creating ChAdOx1.Mix (C-Mix). MetE was omitted due to its size (~82kDa) relative to the other antigens (~10-38kDa), and the tendency of ChAdOx1 to stimulate strong CD8+ T cell responses (33), which had been observed to all antigens except MetE. The expression of each antigen in the C-mix construct was validated (**Supplementary Figure 6**). To synergise with m-EsxI, we utilised ChAdOx1.EsxI (C-EsxI) (19). The mRNA-only regimens were delivered in 2 doses, whilst the ChAdOx1-only groups received a single intranasal dose, time-matched with the booster dose of mRNA-only (**Figure 6A**). Previous research in our group indicated that delivery of ChAdOx1 prior to an RNA-based vaccine may enhance efficacy over the reverse combination (Korompis, M unpublished), and so we proceeded with a heterologous ChAdOx1-mRNA (C-m) combination here. The two doses of this C-m combination regimen were time-matched with the doses of the mRNA-only groups.

In the spleen, heterologous C-m-Mix elicited equivalent CD4+ T cell responses to each antigen, relative to homologous prime-boost with m-Mix (**Figure 6B**) (**Supplementary Figures 7A, B**). CD8+ T cell antigen-specific responses were largely restricted to PPE15 and EspC, with a reduced magnitude in C-m-Mix relative to m-Mix, that was not statistically significant (**Figure 6C**) (**Supplementary Figures 7C, D**). Measurement of IgG endpoint titres to each antigen (**Figures 6D–H**) showed that m-Mix induced high IgG toward EspC and MetE, but variable or low levels of PPE15, ESAT6 and EsxI. Despite the low levels of PPE15-specific IgG in both m-Mix and C-Mix, the C-m-Mix regimen induced a significant titre of IgG, relative to the naïve group (p=0.001). Strong MetE-IgG responses were measured in the sera of m-Mix group, with a trend toward reduced responses in the C-m-Mix group (p=0.1422).

In the EsxI arm of this experiment, two doses of m-EsxI induced strong CD4+ and CD8+ T cell responses, which were significantly greater than in C-EsxI. However, the responses were not significantly different from those in the C-m-EsxI group, which also induced significant CD4+ and CD8+ T cell responses (**Figures 6I, J**) (**Supplementary Figures 7E-H**). Minimal EsxI-specific IgG was observed only in the m-EsxI group (**Figure 6K**).

### 3.7 C-m-Mix vaccination induces antigen-specific mucosal responses

**Figure 7:**
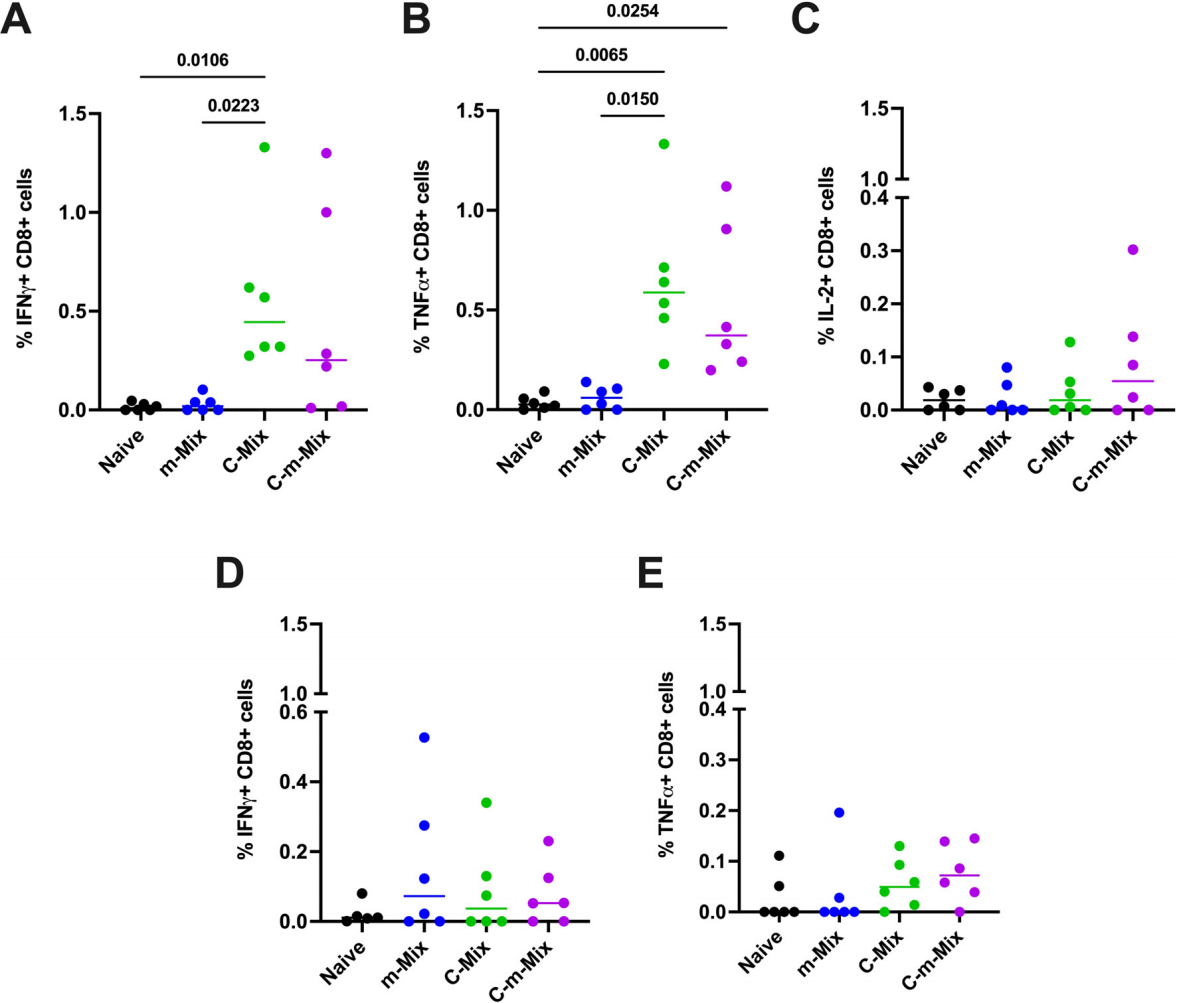
Lung immune responses following vaccination with the C-m-Mix regimen. Groups of CB6F1 mice were vaccinated twice with m-Mix, once with C-Mix, or with one dose of C-Mix and a booster of m-Mix. Four weeks post-boost, immune responses in the lungs of all animals were quantified. **(A, B)** Flow cytometric analysis of PPE15-specific CD8+ T cell **(A)** IFNγ, **(B)** TNFα, and **(C)** IL-2 production in the lungs, or EspC-specific CD8+ T cell **(D)** IFNγ and **(E)** TNFα production in the lungs. Each symbol represents response from 1 animal, n=6 per group. Horizontal bars indicate median. Statistical significance determined via Kruskal-Wallis ANOVA with Dunn’s test for multiple comparisons, selected comparisons displayed only.

Having observed the promising systemic immunogenicity of C-m-Mix, we interrogated the lung response to m-Mix, C-Mix, and C-m-Mix. Importantly, systemic CD4+ and CD8+ T cell responses were similar in this cohort (**Supplementary Figures 8A, B**), as in the prior experiment (**Figures 6B, C**). Given limitations in the number of lung cells available for antigen stimulation, and the proclivity of ChAdOx1 to induce CD8+ T cell responses (33), we investigated the lung response to the two antigens with the greatest CD8+ T cell recognition in the Mix regimens: PPE15 and EspC. A strong PPE15-specific CD8+ T cell response was observed in the C-Mix group, with significantly greater levels of IFNγ and TNFα production than in the naïve or m-Mix groups. These trends were highly similar for C-m-Mix, but only significant for TNFα (**Figures 7A, B**). The C-m-Mix group also trended towards the greatest production of IL-2, but with greater variability (**Figure 7C**). Lower CD8+ T cell responses were seen toward EspC (**Figures 7D, E**), although detectable IFNγ responses were observed in the vaccinated groups, including m-Mix. Similarly, CD8+ T cell production of TNFα in response to EspC demonstrated a trend for greater cytokine production in the C-Mix and C-m-Mix groups. Minimal CD4+ T cell specific lung responses were observed toward either antigen (**Supplementary Figures 8C, D**). Whilst the mRNA platform induces a weak lung response, these data highlight the ability of C-m-Mix to induce both a robust systemic and mucosal response.

### 3.8 Heterologous C-m-Mix vaccination confers strong protection against TB in mice

**Figure 8:**
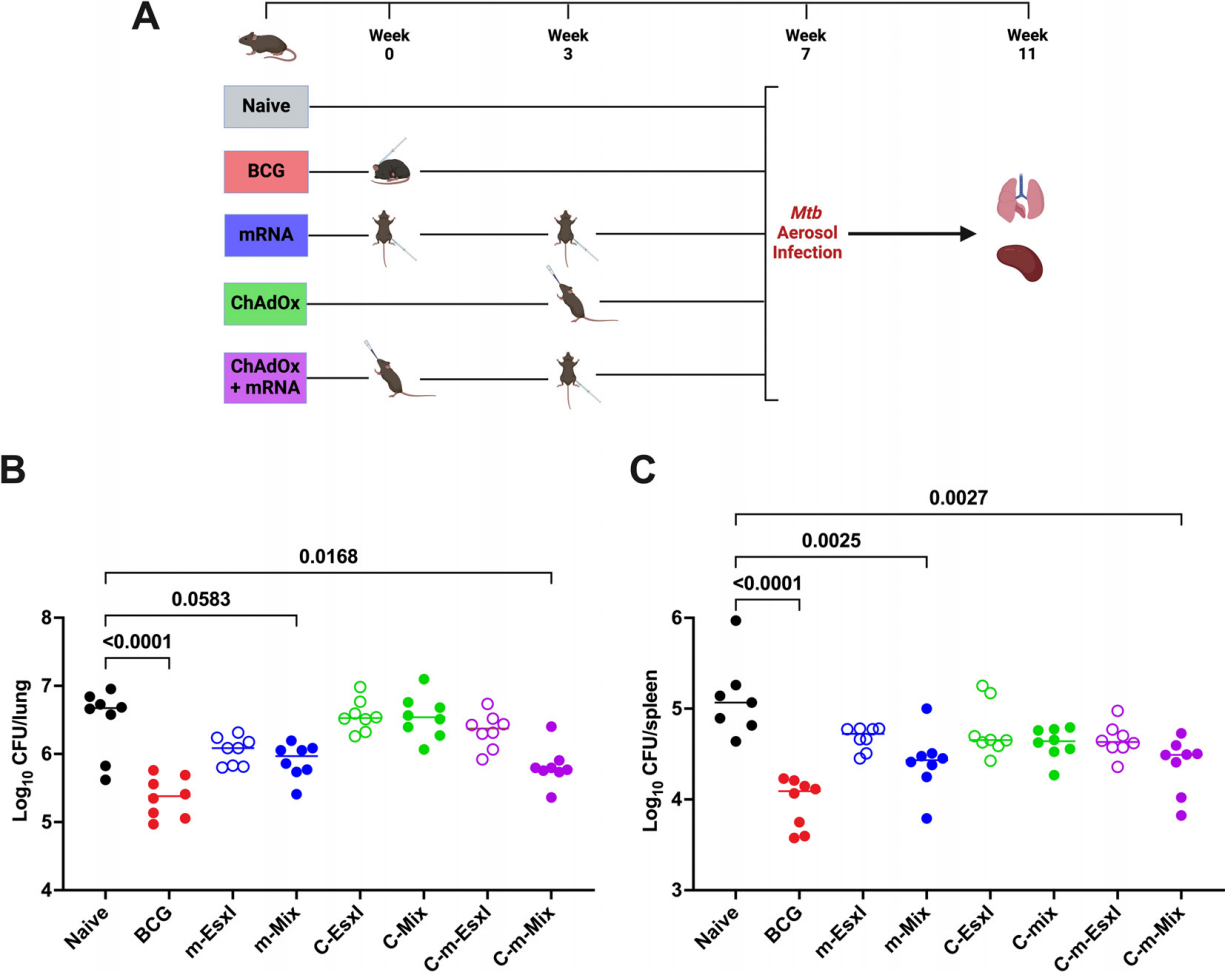
Protective efficacy of heterologous administration of viral vector and mRNA vaccines against TB. **(A)** Immunisation schedule and experimental schematic, created with BioRender.com. BCG-vaccinated mice were infected 7 weeks post-vaccination, whilst all other vaccine groups were infected 4 weeks post-boost. **(B, C)** *Mtb* colony forming units (CFU) in the **(B)** lungs and **(C)** spleen of CB6F1 mice, 4 weeks after challenge with low-dose aerosol *Mtb*. Each symbol represents response from 1 animal, n=8 per group. Horizontal bars indicate median. Statistical significance determined via Kruskal-Wallis ANOVA with Dunn’s test for multiple comparisons, selected comparisons displayed only.

The heterologous C-m regimens were analysed alongside their single platform controls for protection against TB (**Figure 8A**). As seen previously (**Figure 3**), two doses of m-EsxI or m-Mix reduced bacterial burden in the lungs, relative to the unvaccinated cohort, although in this case only approached statistical significance (m-Mix p=0.0583) (**Figure 8B**). There was a small reduction in lung CFU for C-Mix and C-EsxI. Upon heterologous administration of C-m-EsxI, the protection afforded by C-EsxI was only modestly improved. Alternatively, delivery of C-m-Mix resulted in a statistically significant reduction of *Mtb* burden in the lungs (p=0.0168), approaching that of the BCG positive control. In the spleen (**Figure 8C**), all vaccine groups trended towards reduced bacterial burden, relative to the naïve controls. However, only the m-Mix (p=0.0025) and C-m-Mix (p=0.0027) groups conferred statistically significant protection. The lung CFU burden of C-m-Mix compared to both C-Mix and BCG, was also analysed in the Kruskal-Wallis ANOVA, using Dunn’s test for multiple comparisons. The lung bacterial load of C-m-Mix was significantly reduced relative to C-Mix (p=0.0029), and not significantly different to BCG (p=0.3809).

## 4 Discussion

There is an urgent need for a novel TB vaccine. The success of LNP-formulated mRNA against SARS-CoV-2 suggests that application of this promising vaccine platform to intractable pathogens like *Mtb* is a worthwhile pursuit. We characterised 5 novel single antigen mRNA vaccines, as well as a mix regimen, containing all the 5 antigens. We demonstrated the potent immunogenicity of these six vaccines, across multiple cellular and humoral measures, with two regimens, m-EsxI and m-Mix, affording significant protection against TB in the murine model. Whilst the ability of this platform to boost a protective effect of BCG is not yet clear, further work indicated a clear potential of the m-Mix to synergise with heterologous viral vector platforms to increase protective efficacy.

Thorough immunological analysis has demonstrated that the mRNA vaccine platform consistently induces potent humoral responses (34–36), which can be influenced by LNP composition – particularly the ionizable lipid component (37). In this study, m-PPE15, m-EspC, and m-MetE induced high sera IgG titres. Conversely, the lack of significant IgG towards m-ESAT6 and m-EsxI presents an interesting dimension for analysis, given neutralising antibody responses are commonly a correlate of protection against the viral pathogens targeted by mRNA vaccines (38). An apparent reason for the lack of antibody production seen towards ESAT6 and EsxI, is the absence of proper protein folding upon translation, which would still enable the significant cellular response towards both antigens. The two proteins are small (~10kDa), relatively hydrophobic, and ESAT6 requires binding to its partner, CFP-10, for conformational stability (39). Given EsxI (ESAT6-like protein 1) is the result of an evolutionary ESAT6 duplication and has its own adjacent probable binding partner (EsxJ) (40), it is likely that both ESAT6 and EsxI were similarly incapable of correct folding after translation *in vivo*.

Functional characterisation of mRNA vaccines has also regularly reported induction of protective cellular immunity (41–43), which remains a central tenet of TB vaccine design. Here, we demonstrate that 4 of the 5 promising single mRNA antigens induce a strong T_H_1 response, in the form of antigen-specific CD4+ T cells producing IFNγ, TNFα, or IL-2. These factors have long been considered wholly indispensable in TB protection (44–47). In addition, we show that these vaccines induce robust levels of CD4+ IFNγ+ TNFα+ IL-2+ T cells. The enhanced effector functions of these polyfunctional cells are proposed to be associated with protection, although this correlation is yet to be fully proven (48). Whilst we did not observe consistent induction of IL-17 in this study, a cytokine predicted to be involved in vaccine-mediated protection against *Mtb* (49, 50), future work will reveal whether this is an inherent characteristic of the mRNA platform, or due to specific antigens tested here. CD8+ T cells also play a significant role in immunity against *Mtb*, particularly when in concert with T_H_1 immunity (51, 52). In this study, we show strong and consistent CD8+ T cell responses to PPE15, EspC, and EsxI.

In keeping with the cellular dogma of TB vaccine design, it is m-EsxI, which induces the greatest CD8+ T cell response of any single antigen vaccine and a strong CD4+ T cell response, that is the only single construct to achieve statistically significant protection against *in vivo Mtb* infection, albeit in only one of the two relevant infection studies. Given the lack of IgG specific to EsxI, it can be postulated that the mechanism of anti-mycobacterial immunity by m-EsxI is cellular. However, the lack of EsxI-specific response in the m-Mix draws into question the protective mechanism for that regimen. Whilst m-EspC and m-MetE induce high titres of specific IgG in m-Mix, those single antigen vaccines do not show efficacy against *Mtb* infection. This is despite the fact that both EspC and MetE have demonstrated protection previously as single antigens, when delivered as high antibody-inducing protein-adjuvant vaccines (Stylianou et al, in preparation) (Almujri, S, et al. in preparation). It is possible that the combination of the EspC- and MetE-specific IgG titres assist in the protection shown by m-Mix, but further work is needed to deconvolute their roles. Examination of the m-Mix cellular response points to two alternate possible factors as responsible for the protective effect: (1) the greater breadth of antigen-specific cellular responses in m-Mix (strong CD4+ T cell response to PPE15, ESAT6, MetE, and strong CD8+ T cell response to PPE15, EspC) and (2) the increased PPE15-specific CD8+ T cell response, which rises to a magnitude equivalent to that towards EsxI, in the protective m-EsxI vaccine. The fact that the cellular responses are likely responsible for the protection of m-EsxI, and recognition of multiple antigens is likely important for the protection of m-Mix, provide strong indications for the future direction of TB mRNA vaccine design.

The interesting changes in PPE15- and EsxI-specific responses in m-Mix, relative to the single antigen constructs, were shown not to be due to the decreased dose of each vaccine utilised in m-Mix. However, the trends prompt a question of potential interactions occurring between the antigens upon mixing. In *Mtb*, *esxI* and *ppe15* belong to the ESX-5a gene cluster: a type VII secretion system (40). The constituents of ESX-5a (*esxI, esxJ, ppe15*, and *pe8*) are predicted to be secreted by ESX-5 structural proteins located elsewhere in the genome, so no direct contact between EsxI and PPE15 has been established, aside from genomic proximity and secretion by ESX-5a. Potentially, the large hydrophobic region of PPE15 that binds its partner, PE8 (53), could be facilitating a non-specific interaction *in vivo* with the hydrophobic region of EsxI, which is likely involved in binding EsxJ. This might also explain the abrogation of the PPE15-specific IgG response in m-Mix, relative to m-PPE15, as an EsxI binding partner could alter the available tertiary structure of PPE15 for antibody binding. Although the use of a 5-antigen fusion construct might address these trends, it could also alter the tertiary structure of each antigen and relatively reduce the molar expression of smaller antigens in the fusion, versus in m-Mix. Investigations elsewhere have demonstrated largely equivalent antigen-specific immunogenicity and efficacy when mixing 20 separate influenza mRNA constructs in a single delivery (54), when delivering co-encapsulated quadrivalent influenza mRNA constructs (55), or when delivering a co-encapsulated mRNA triplex against Coronavirus species (56). These results suggest that the immunological trends of m-Mix seen in our study may be specific to the antigens selected here, and that further investigation is warranted.

Whilst the ability of the mRNA platform to maintain strong immunogenicity when applied as a boost to BCG is promising, the lack of enhanced protection above that afforded by BCG presents an important hurdle to overcome. However, the result may reflect the stringency of the murine model utilised here, rather than an inability of the mRNA platform to achieve the outcome. Low-dose aerosol delivery of <100 CFU *Mtb* is widely utilised in mouse models of TB, given it consistently infects all animals exposed. However, ultra-low doses (1-3 CFU) may more closely mimic natural infection and allow greater resolution for protective effects of vaccine candidates (57), rather than potentially overwhelming the lung immune microenvironment (58). Future histopathological analysis of lung tissue inflammation may also provide further insight into the ability of the mRNA platform to protect against *Mtb* infection. It is particularly notable that CB6F1 mice have greater genetic diversity and natural resistance to *Mtb* infection (59) than their commonly used parental strains (C57BL/6 x BALB/c); potentially masking vaccine effects visible elsewhere. Whilst the stringency of this mouse model was consciously chosen to select the most effective vaccine candidates for progression, CB6F1 are also very sensitive to protection afforded by BCG. CB6F1 mice require ten-fold lower doses of BCG than BALB/c mice to be protected against *Mtb* infection, and BCG doses as low as 30 CFU demonstrate significant protection (60). This responsiveness to BCG may explain why m-Mix and m-EsxI protected mice vaccinated only with mRNA, but these effects were masked in the presence of BCG, despite the persistence of very strong immunogenicity of the mRNA candidate vaccines. Further work, that may include variation of the infectious dose, or utilisation of mice models like C3HeB/FeJ which display greater sensitivity to *Mtb* infection (61), could help uncover whether the lack of BCG boosting by mRNA is simply due to a strong BCG effect in the current mouse model.

Mucosal administration of TB vaccines has been the subject of recent interest, given the potential for this route to induce resident memory at the point of natural *Mtb* infection (62, 63). At this time, LNP-formulated mRNA vaccines have not been translated for clinical delivery to the lung (64), although preclinical development of novel LNPs for this purpose is underway (65, 66), and intranasal delivery of naked mRNA has demonstrated efficacy against *Mtb* infection (17). Notably, it has been suggested that parenterally administered SARS-CoV-2 mRNA vaccines were effective at preventing severe COVID-19 due to their strong systemic immunity but failed to consistently prevent disease transmission due to variable stimulation of mucosal immunity and production of mucosal IgA (67, 68). This presents a valid concern for preventing pulmonary TB infection by parenteral mRNA vaccination, and characterisation of the ability of our promising mRNA vaccines to induce lung tissue resident memory responses is the subject of future work, given these cells have previously been shown to be associated with vaccine-mediated protection in mice (18). In the current study, we utilised intranasal delivery of the ChAdOx1 viral vector platform to specifically target mucosal immunity, in heterologous combination with the strong systemic immunity of our novel mRNA vaccines. We show that the C-m-Mix combination had equivalent lung CD8+ T cell responses to C-Mix, which exceeded that of m-Mix, whilst also largely matching the spleen CD4+ and CD8+ T cell responses, and sera IgG titres, of m-Mix. Fittingly, upon *Mtb* challenge C-m-Mix conferred the greatest overall protection. These results are in accord with multiple studies that have highlighted the increased immunogenicity and efficacy of heterologous ChAdOx1 prime and mRNA boost against SARS-CoV-2, albeit with parenteral delivery of the viral vector (69–71). Whilst our study is the first to apply such mRNA platforms to TB, elsewhere a heterologous regimen of a novel replicating RNA platform with the candidate protein-adjuvant vaccine, ID91, synergised to provide significant protective efficacy against TB (16). Similarly, and in keeping with the C-m order of delivery used in this study, an intranasal viral vector prime and DNA vaccine boost induced superior cellular immunity and protective efficacy against *Mtb* infection than the reverse order (72). Moreover, ChAdOx1 boosted by modified Vaccinia virus Ankara (MVA) conferred superior protection against TB, than the alternate MVA-ChAdOx1 prime-boost (73). The prime-boost order of viral vector-subunit vaccine has demonstrated efficacy against malaria (74) and HIV (75). However, it is noteworthy that studies of other preclinical TB vaccines have shown abrogation of specific CD8+ T cell responses and even long-term protection against *Mtb*, if the order of a protein-adjuvant prime and respective DNA (76) or viral vector (77) boost is reversed. As such, further investigation is needed to determine whether an m-C-Mix regimen may display different efficacy than C-m-Mix. This is pertinent as m-C-Mix may have the potential to elicit a “prime-pull” effect (78), in which the ChAdOx1 vector draws the very strong mycobacterial-specific CD8+ T cell responses, induced by parenteral mRNA prime, into the lung compartment, thus further increasing efficacy against *Mtb* infection.

Whilst the results of this study demonstrate great promise for future application of mRNA technology to a TB vaccine, potential issues linger. Currently, very few clinical trials are examining the efficacy of mRNA vaccines against bacterial pathogens (BioNTech for TB (NCT05547464), Moderna, Inc. for Lyme disease (NCT05975099)). It is yet to be determined whether these will show the same clinical success as mRNA against viral pathogens. Moreover, recommendations for mRNA vaccine storage regularly require temperatures below freezing, presenting a major concern for areas lacking cold chain capabilities (79); a particularly relevant point for TB. Current clinical trials have found success with refrigerator stable mRNA vaccines (Moderna, Inc. NCT05815498), although thermostable TB vaccines would be ideal (80). TB disease distribution is also a factor when considering the longevity of mRNA-induced immune responses and reports of waning immunity (81–83), given the difficulty of reaching certain populations for booster vaccinations. Long term efficacy studies of mRNA vaccines are necessary to address this concern.

The results of this study are the first to present current generation mRNA vaccine technology as a promising platform for protection against TB. Our data supports further investigation of the m-Mix regimen, as well as providing support for development of the heterologous C-m-Mix regimen, in the effort for global TB eradication.

## Data Availability

The raw data supporting the conclusions of this article will be made available by the authors, without undue reservation.
