# Supplementary material for: Novel mRNA vaccines induce potent immunogenicity and afford protection against tuberculosis
Source: Front Immunol. 2025 Feb 13;16:1540359. doi: 10.3389/fimmu.2025.1540359 (PMC11865049; doi:10.3389/fimmu.2025.1540359)
Supplement: Supplementary file 1 [file DataSheet1.pdf]

Supplementary Information

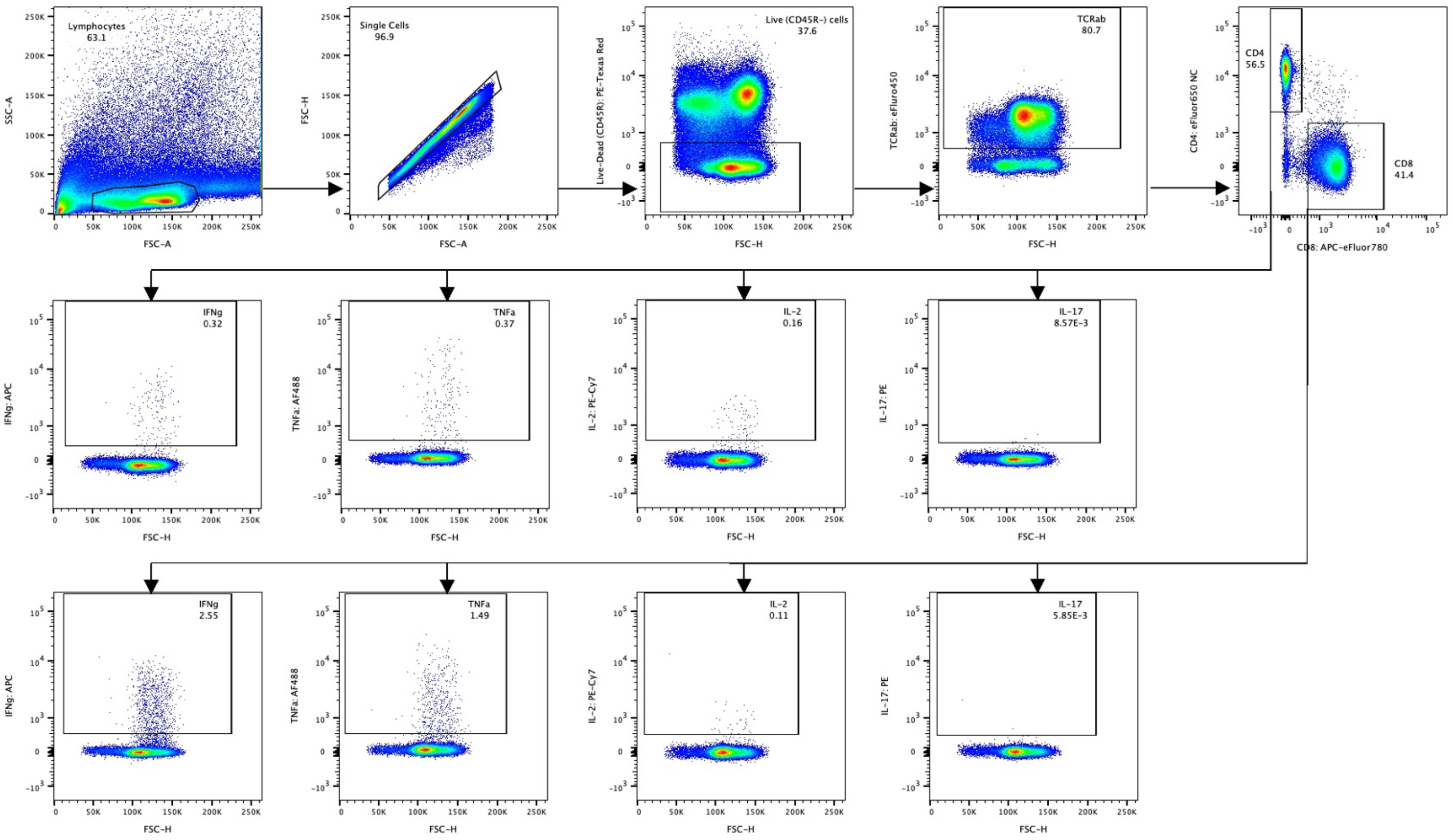

Supplementary Figure 1. Gating strategy for flow cytometry. In conjunction with intracellular cytokine staining (ICS), this hierarchical strategy was used to quantify antigen-specific responses in mouse splenocytes and lung cells. At each experimental endpoint, cell suspensions were isolated from tissue, stimulated for 6 hours with a relevant antigen, and then stained for quantification of IFNγ, TNFα, IL-2, and IL-17 production, by CD4+ and CD8+ T cells.

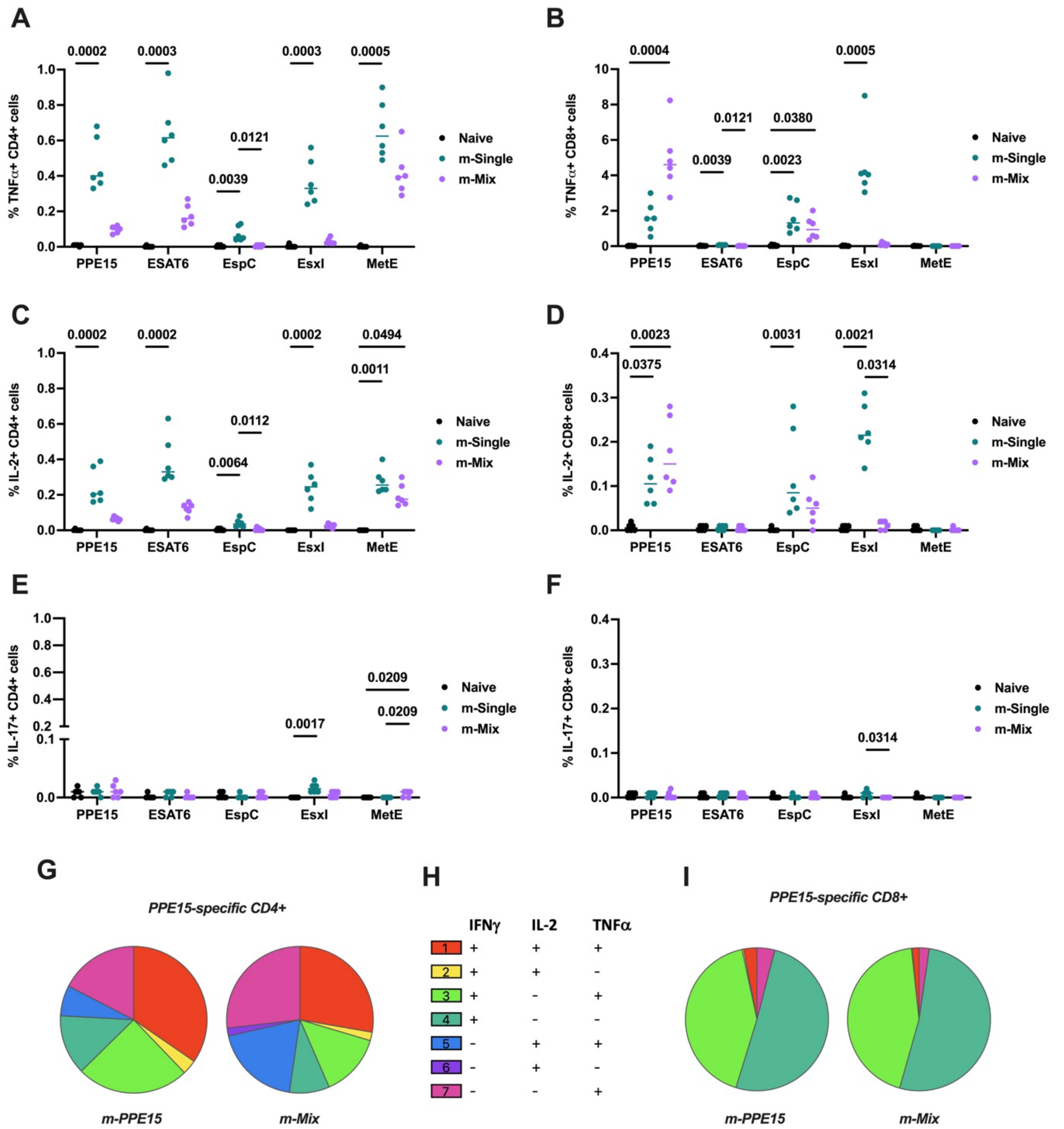

	IFNγ	IL-2	TNFα
1	+	+	+
2	+	+	-
3	+	-	+
4	+	-	-
5	-	+	+
6	-	+	-
7	-	-	+

Supplementary Figure 2. Antigen-specific immunogenicity of novel TB mRNA vaccines. Groups of CB6F1 mice were vaccinated twice with one of five single antigen mRNA vaccines (m-Single, 5 µg per dose) or an equal mix of all 5 antigens (m-Mix, 1 µg per antigen, 5 µg total dose). Immune responses in the spleen were quantified four weeks post-boost. (A-F) Flow cytometric analysis of CD4+ T cell production of (A) TNFα, (C) IL-2, and (E) IL-17, or CD8+ T cell production of (B) TNFα, (D) IL-2, and (F) IL-17, in response to stimulation by relevant antigens listed on x-axis. (G-I) Relative proportions of multifunctional cytokine-secreting (G) CD4+ or (I) CD8+ T cells, specific to PPE15, with (H) legend indicating relevant cytokine secretion of each population. PPE15 multifunctional response is shown as a representative example of other antigens, that also have detectable polyfunctional responses. Each symbol represents response from 1 animal, n=6 per group. (A-F) Horizontal bars indicate median. Statistical significance was determined via Kruskal-Wallis ANOVA with Dunn's test for multiple comparisons.

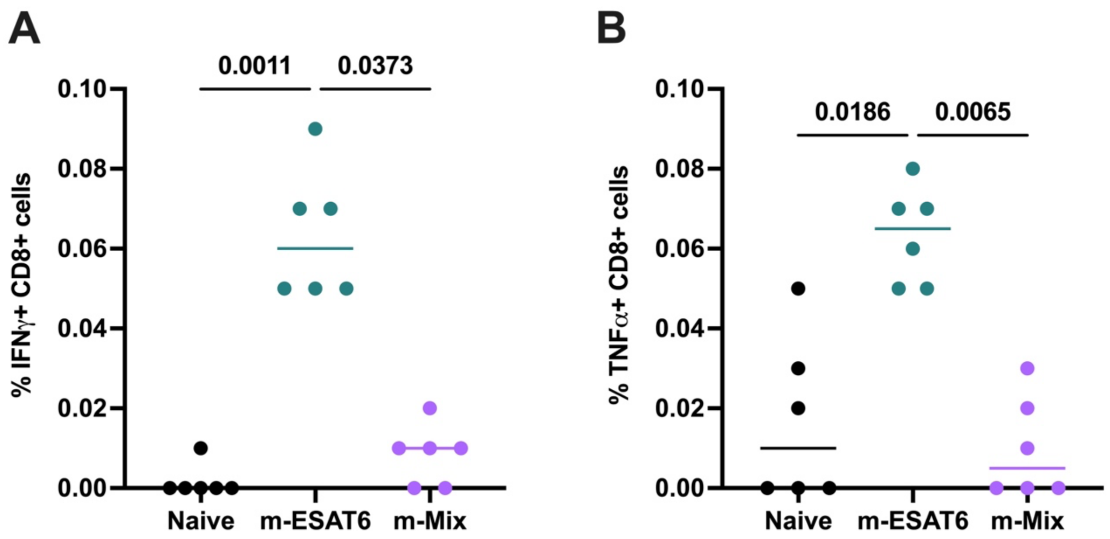

Supplementary Figure 3: ESAT6-specific CD8+ T cell immunogenicity. These results are the same as those shown for ESAT6 in Figure 1C and supplementary figure 2B, but magnified here for clarity. Groups of CB6F1 mice were vaccinated twice m-ESAT6 or m-Mix (5µg doses). Immune responses in the spleen were quantified four weeks post-boost. (A-B) Flow cytometric analysis of CD8+ T cell production of (A) IFNγ and (B) TNFα in response to stimulation with an overlapping ESAT6 peptide pool. Each symbol represents response from 1 animal, n=6 per group. Horizontal bars indicate median. Statistical significance was determined via Kruskal-Wallis ANOVA with Dunn's test for multiple comparisons.

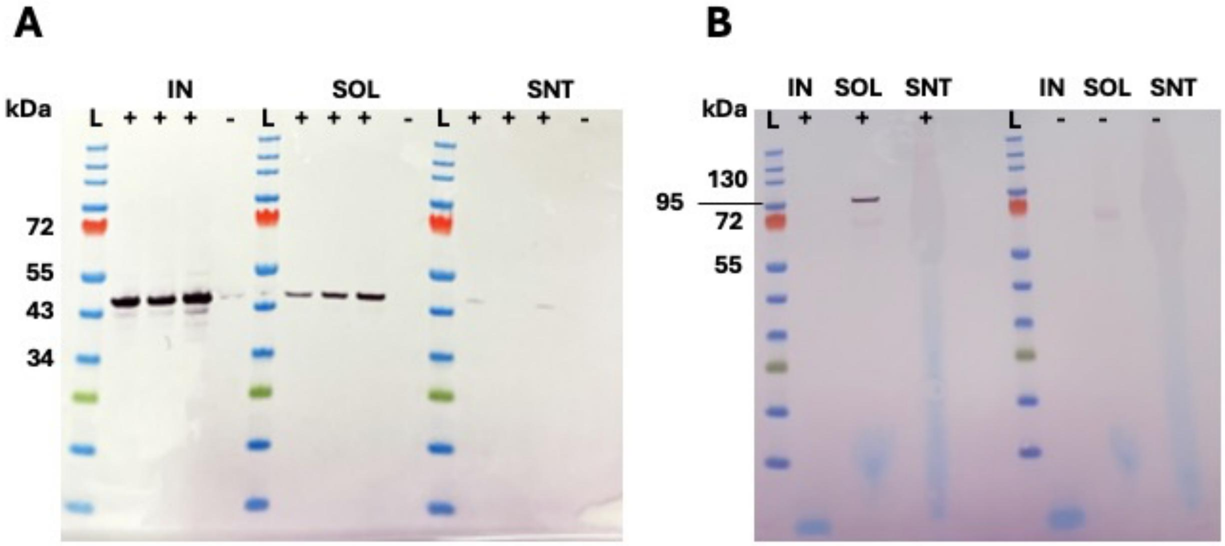

Supplementary Figure 4. *in vitro* expression of PPE15 and MetE mRNA. Western blotting of HEK293 cell insoluble fraction (IN), soluble fraction (SOL) and culture supernatant (SNT), following 24 hours of transient transfection with unformulated mRNA encoding (A) PPE15 or (B) MetE. Transfection was facilitated by Lipofectamine 2000. Positive signs indicate transfected cells (conducted in biological triplicate for PPE15), whilst negative signs indicate negative control cells. Blots were probed with (A) anti-PPE15 or (B) anti-MetE sera from female mice vaccinated with respective purified protein in commercial adjuvant. This experimental procedure was repeated for ESAT6, EsxI, and EspC, but no expression was detected (blots not shown).

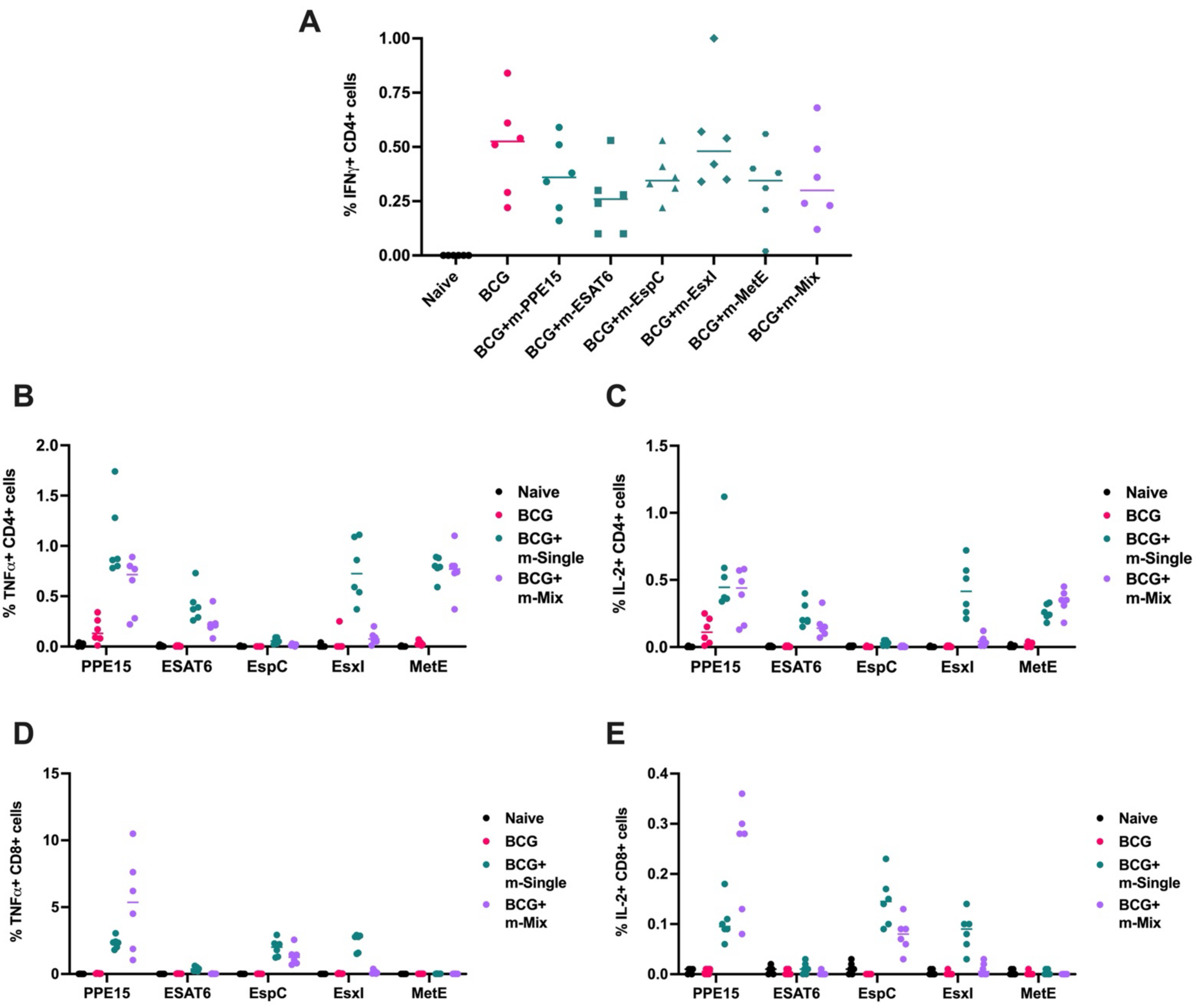

Supplementary Figure 5. Evaluation of TB mRNA vaccine immunogenicity when applied as a boost to BCG. Groups of CB6F1 mice were vaccinated with BCG, and after 10 weeks rest, relevant groups were vaccinated twice with single antigen mRNA vaccines (m-Single) or an equal mix of all 5 antigens (m-Mix). Four weeks post-boost, immune responses in the spleen of all animals were quantified. (A) Flow cytometric analysis of CD4+ T cell IFNγ expression in response to PPD stimulation. (B-E) Flow cytometric analysis of CD4+ T cell production of (B) TNFα and (C) IL-2, or CD8+ T cell expression of (D) TNFα and (E) IL-2 in response to antigen stimulation listed on x-axis. Each symbol represents response from 1 animal, n=6 per group. Horizontal bars indicate median.

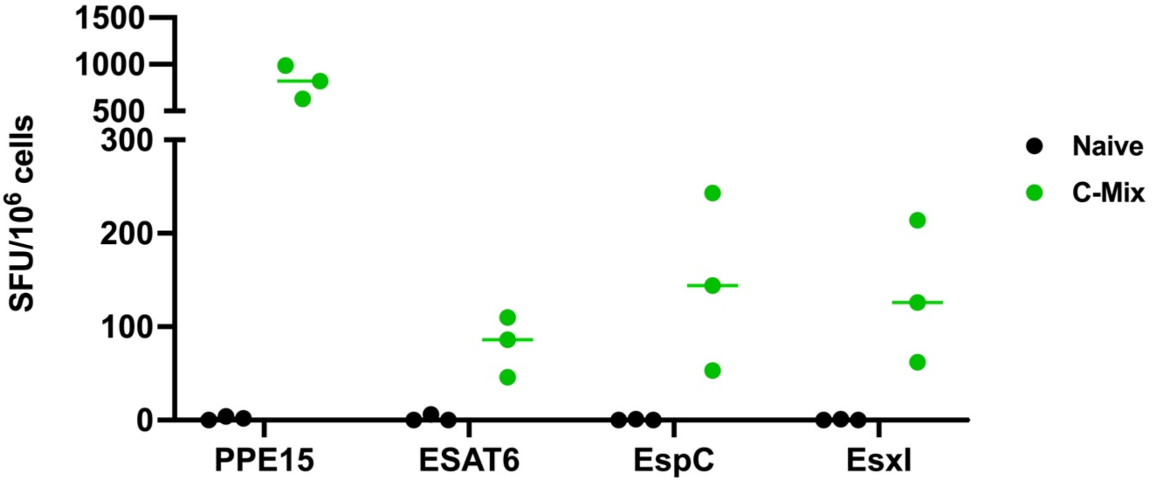

Supplementary Figure 6. Immune recognition of each antigen in 15-3E construct delivered by C-Mix. Antigen-dependent IFNγ responses were assessed by ELISpot, 4 weeks after intramuscular vaccination with 10^8 ifu of C-Mix. X-axis indicates overlapping peptide pools of antigen used for stimulation. Data is presented as spot-forming units (SFU) per 1 million cells (SFU/10^6 cells). Each symbol represents response from 1 animal, n=3 per group. Horizontal bars indicate median.

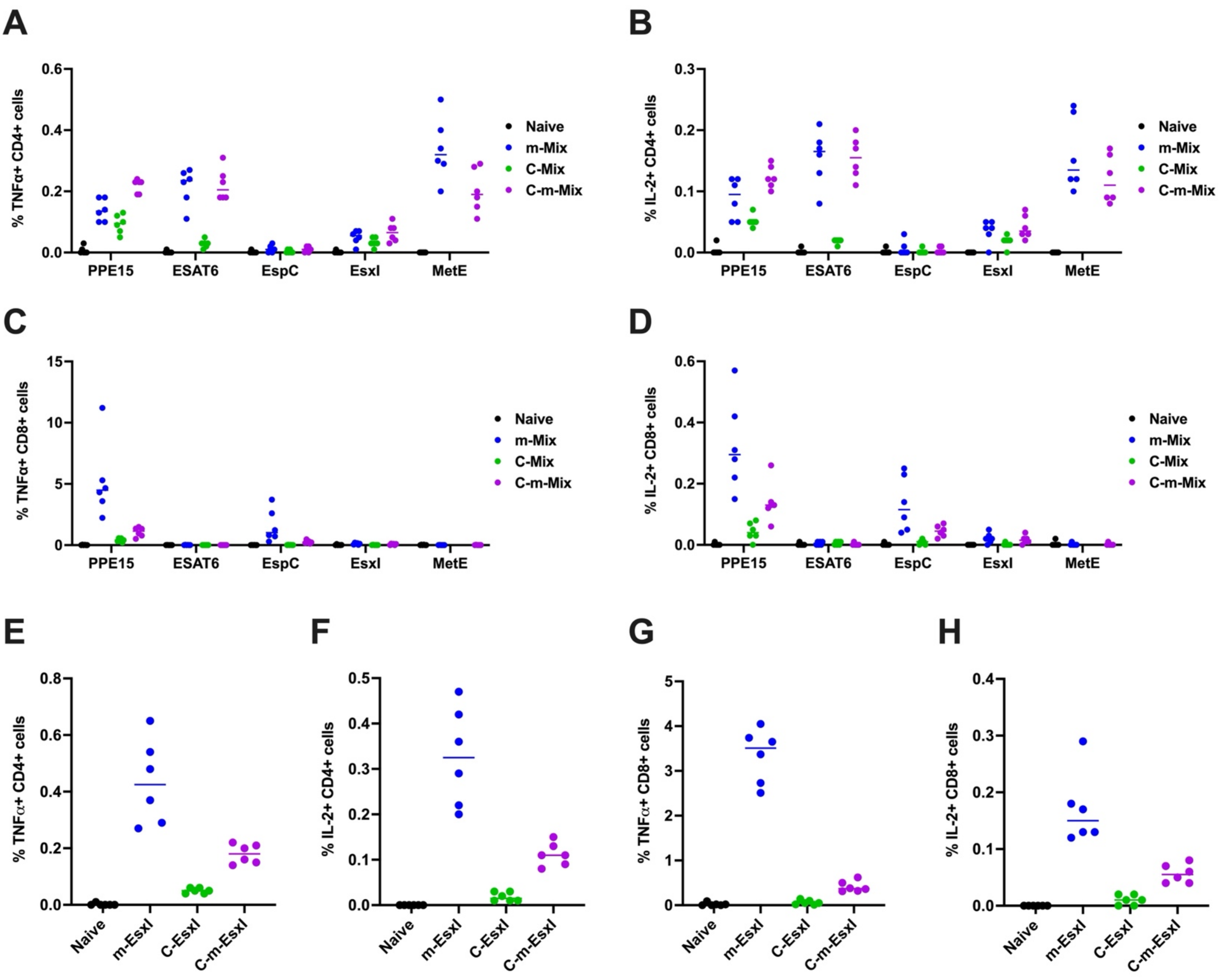

Supplementary Figure 7. Immune responses following heterologous ChAdOx-mRNA prime-boost. (A-D) Flow cytometric analysis of CD4+ T cell production of (A) TNFα and (B) IL-2, or CD8+ T cell production of (C) TNFα and (D) IL-2, in response to stimulation by antigens listed on x-axis, for animals of the Mix experimental arm. (E-H) Flow cytometric analysis of CD4+ T cell production of (E) TNFα and (F) IL-2, or CD8+ T cell production of (G) TNFα and (H) IL-2, in response to stimulation EsxI. Each symbol represents response from 1 animal, n=6 per group. Horizontal bars indicate median.

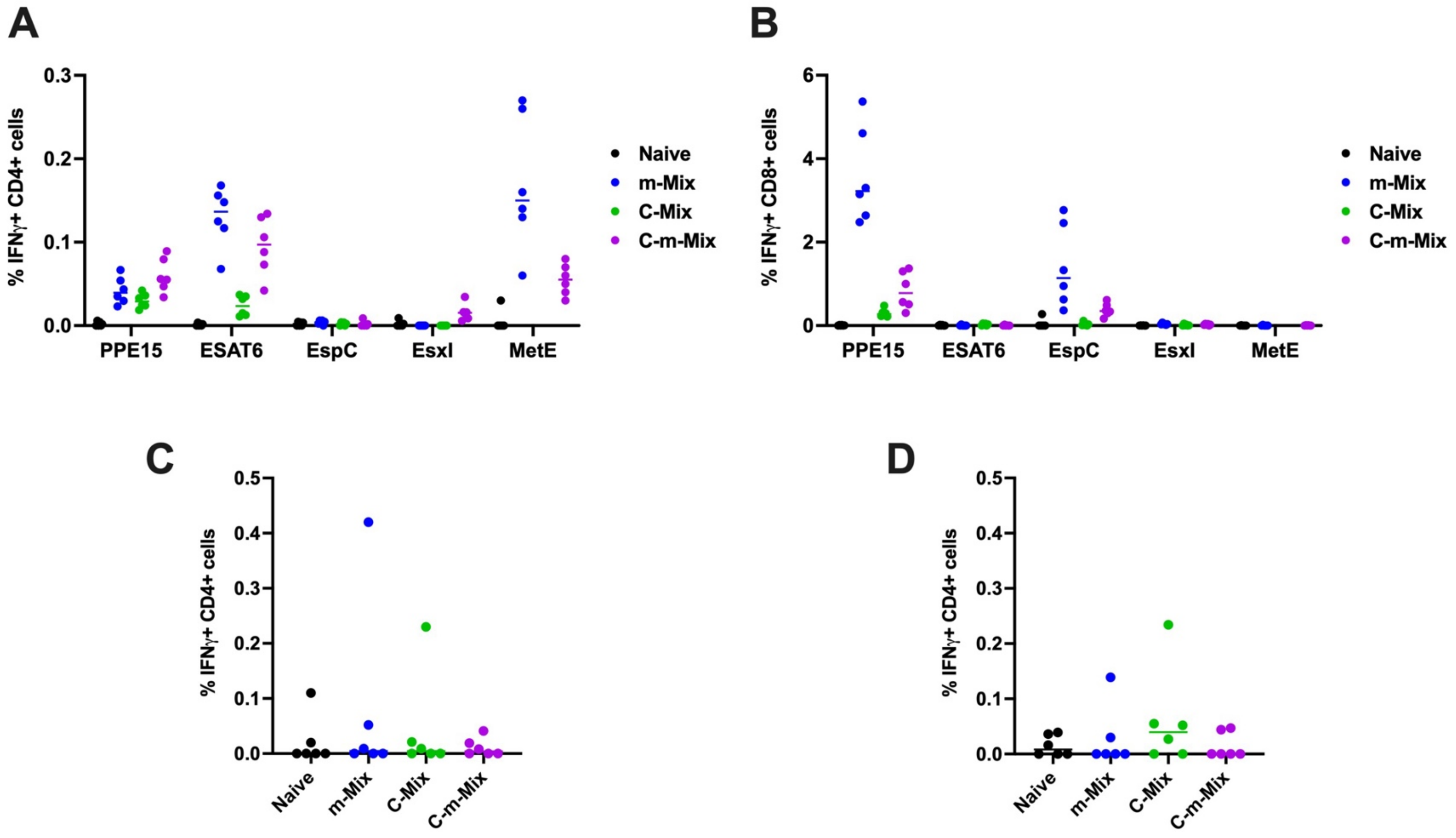

Supplementary Figure 8. Systemic immune responses following heterologous ChAdOx-mRNA prime-boost. Groups of CB6F1 mice were vaccinated twice with m-Mix, once with C-Mix, or with C-m-Mix. Four weeks post-boost, immune responses in the spleen and blood of all animals were quantified. (A-B) Flow cytometric analysis of IFNγ expression by (A) CD4+ or (B) CD8+ T cells in the spleens of animals in Mix groups, in response to stimulation by relevant antigens listed on x-axis. (C-D) Flow cytometric analysis of IFNγ expression by CD4+ T cells in the lungs of animals in Mix groups, in response to stimulation by (A) PPE15 or (B) EspC. Each symbol represents response from 1 animal, n=6 per group. Horizontal bars indicate median.
