# Supplementary material for: Novel mRNA vaccines induce potent immunogenicity and afford protection against tuberculosis
Source: Front Immunol. 2025 Feb 13;16:1540359. doi: 10.3389/fimmu.2025.1540359 (PMC11865049; doi:10.3389/fimmu.2025.1540359)
Supplement: Supplementary file 2 [file Table1.pdf]

Supplementary Table 1. Tabulated summary of the *in vitro* expression, and immunogenicity, of each antigen utilised in this study.

		**Antigen-specific responses as m-Single vaccine (5µg antigen)**			
**Antigen**	**Expression during *in vitro* transfection**	**CD4+ T cell**	**CD8+ T cell**	**IgG Titres**	**Response changes as part of m-Mix vaccine (1µg antigen)**
PPE15	Detected	High	High	High	Increase in CD8+ T cell response, significant reduction in IgG titres
ESAT6	Not detected	High	Low	Not detected	Non-significant
EspC	Not detected	Low	High	High	Non-significant
EsxI	Not detected	High	High	Not detected	Large decrease in CD4+ and CD8+ T cell responses
MetE	Detected	High	Not detected	High	Non-significant
